# Piperlongumine regulates epigenetic modulation and alleviates psoriasis-like skin inflammation via inhibition of hyperproliferation and inflammation

**DOI:** 10.1038/s41419-019-2212-y

**Published:** 2020-01-10

**Authors:** Sowjanya Thatikonda, Venkatesh Pooladanda, Dilep Kumar Sigalapalli, Chandraiah Godugu

**Affiliations:** 1 Department of Regulatory Toxicology, National Institute of Pharmaceutical Education and Research (NIPER), Balanagar, Hyderabad, Telangana 500037 India; 2 Department of Medicinal Chemistry, National Institute of Pharmaceutical Education and Research (NIPER), Balanagar, Hyderabad, Telangana 500037 India

**Keywords:** Cell death and immune response, Psoriasis

## Abstract

Psoriasis is an autoimmune skin disease, where chronic immune responses due to exaggerated cytokine signaling, abnormal differentiation, and evasion of keratinocytes apoptosis plays a crucial role in mediating abnormal keratinocytes hyperproliferation. From the therapeutic perspective, the molecules with strong anti-proliferative and anti-inflammatory properties could have tremendous relevance. In this study, we demonstrated that piperlongumine (PPL) treatment effectively abrogated the hyperproliferation and differentiation of keratinocytes by inducing ROS-mediated late apoptosis with loss of mitochondrial membrane potential. Besides, the arrest of cell cycle was found at Sub-G1 phase as a result of DNA fragmentation. Molecularly, inhibition of STAT3 and Akt signaling was observed with a decrease in proliferative markers such as PCNA, ki67, and Cyclin D1 along with anti-apoptotic Bcl-2 protein expression. Keratin 17 is a critical regulator of keratinocyte differentiation, and it was found to be downregulated with PPL significantly. Furthermore, prominent anti-inflammatory effects were observed by inhibition of lipopolysaccharide (LPS)/Imiquimod (IMQ)-induced p65 NF-κB signaling cascade and strongly inhibited the production of cytokine storm involved in psoriasis-like skin inflammation, thus led to the restoration of normal epidermal architecture with reduction of epidermal hyperplasia and splenomegaly. In addition, PPL epigenetically inhibited histone-modifying enzymes, which include histone deacetylases (HDACs) of class I (HDAC1–4) and class II (HDAC6) evaluated by immunoblotting and HDAC enzyme assay kit. In addition, our results show that PPL effectively inhibits the nuclear translocation of p65 and a histone modulator HDAC3, thus sequestered in the cytoplasm of macrophages. Furthermore, PPL effectively enhanced the protein–protein interactions of HDAC3 and p65 with IκBα, which was disrupted by LPS stimulation and were evaluated by Co-IP and molecular modeling. Collectively, our findings indicate that piperlongumine may serve as an anti-proliferative and anti-inflammatory agent and could serve as a potential therapeutic option in treating psoriasis.

## Introduction

Psoriasis is a complex immune-mediated hyperproliferative disease characterized by excessive growth of epidermal keratinocytes, which form elongated rete ridges protruding into the dermis with aberrant terminal differentiation^1^. The vital triggers implicated in keratinocyte abnormal responses are associated with the activation of the cellular immune system, which primarily includes T cells, macrophages, dendritic cells, and endothelial cells with a broad network circuitry of growth factors like epidermal growth factor (EGF), insulin-like growth factor, vascular endothelial growth factor, and keratinocyte growth factor, along with cytokines such as interleukins (ILs; IL-1, IL-6, IL-17, IL-19, IL-20, and IL-22), tumor necrosis factor-α (TNF-α), and interferons (IFNs), which further regulate the keratinocyte proliferation^2–5^. On the other hand, keratinocytes from psoriatic lesions exhibit increased resistance to apoptosis, which concurs to the thickened epidermis. The microarray transcriptome analysis in psoriatic lesions revealed upregulated anti-apoptotic and downregulated pro-apoptotic genes expression^6^.

The growing evidence suggests that signal transducer and activator of transcription 3 (STAT3) and p65, a subunit of nuclear factor kappa-light-chain-enhancer of activated B cells (NF-κB p65), have a pivotal role in mediating the positive feedback loop in psoriasis by translocating to the nucleus and stimulate transcription of proliferation and inflammation-regulated genes expression. In parallel, sustained activation of the anti-apoptotic phosphatidylinositol 3-kinase/protein kinase B (PI3K/AKT) and mitogen-activated protein kinases (MAPKs), which includes extracellular signal–regulated kinase 1/2 (ERK1/2), c-Jun N-terminal kinase (JNK), and p38 signaling pathways contribute to aberrant expression of keratin 17 (K17)^7–9^.

An important epigenetic phenomenon in macrophages is the transient inflammatory stimulus-mediated changes that include histone deacetylase (HDAC) activation, which increases the production of pro-inflammatory cytokines in wide disease models, including septic shock, acute respiratory distress syndrome, renal fibrosis, and rheumatoid arthritis, where abrogation of HDAC activity reduced inflammatory cytokines^10–12^. Moreover, HDAC inhibition was found to suppress the polarization of T helper type 17 (Th17) cells and STAT3 inhibition^13^, while HDAC3 inhibition reduced TNF-α, with a concomitant increase in the acetylation of p65, which has been suggested to play a key role in attenuating IκΒα-mediated NF-κΒ transcriptional activity^14–16^.

The present traditional treatments for treating psoriasis include psoralen and ultraviolet A therapy, methotrexate, retinoids, and Tacrolimus (TAC), which are effective but exhibit several shortcomings, including inconvenience and toxicity with long-term usage^17^. Hence, there is a need for the development of effective and safer alternatives. Imiquimod (IMQ) is a toll-like receptor 7/8 ligand activator that are expressed by monocytes, macrophages, and plasmacytoid dendritic cells, thereby the production of pro-inflammatory cytokines and chemokines increases aberrantly, with profound Th1 and Th17 responses, and these events lead to the direct influx of immune cells to the site of IMQ application, thereby inducing inflamed scaly skin lesions^18,19^. In the present study, for the experimental design, IMQ-induced psoriasis model was used as this model is rapid, reproducible, and closely recapitulates human psoriasis in terms of histopathological alterations and cellular infiltrates. Piperlongumine (PPL) is a chief constituent of the fruit from Long pepper (*Piper longum*), which is considered to be the “historical spice as a future medicine” and has been extensively studied in cancer treatment and in treating various ailments, including rheumatoid arthritis, osteoarthritis, asthma, neurogenerative diseases, diabetes, melanogenesis, lupus nephritis, and hyperlipidememia^14,20^, with little systemic toxicity^21^. Hence, this study aimed to investigate anti-proliferative and anti-inflammatory effects of PPL on keratinocytes and macrophages and its involvement in epigenetic modulation and associated signaling mechanisms, which could be effective against psoriasis.

## Results

### PPL reduces IMQ-induced psoriasis-like skin inflammation and normalizes the epidermal architecture

**Fig. 1:**
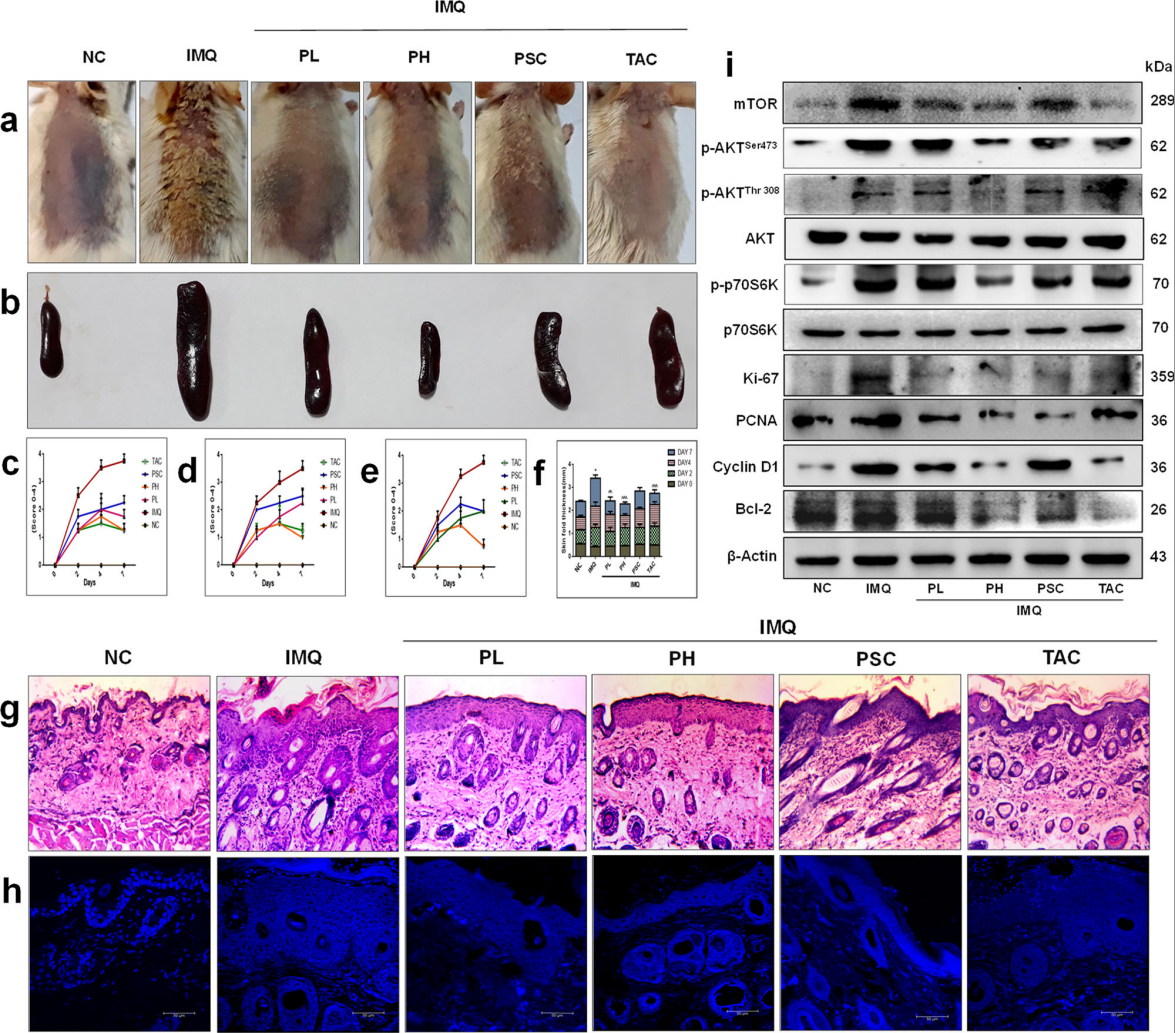
Piperlongumine (PPL) intervention attenuates Imiquimod (IMQ)-induced psoriasis severity in BALB/c mice. Mice were received IMQ, PPL, and Tacrolimus (TAC). **a** After the completion of study, mice were anesthetized and mages of IMQ-induced hyperplasia on mouse back skin with and without PPL treatment were taken by digital camera. **b** PPL attenuated splenomegaly induced by topical IMQ. PASI scoring was recorded on days 0, 2, 4, and 7 and the cumulative scores of **c** redness, **d** scaling, and **e** thickness were measured. **f** Skin fold thickness in all groups was measured by Vernier calipers. Representative histological sections of the back skins of mice where **g** H & E and **h** DAPI staining show reduced epidermal hyperplasia and parakeratosis induced by IMQ, and **i** western blotting analysis was performed with the indicated antibodies, where PPL inhibits IMQ-induced Akt pathway-, apoptosis-, and proliferation-mediated protein expression. The data represent mean ± SD (*n* = 5 mice per group). *P < 0.05 vs. NC group. ^^*P* < 0.01 and ^^^*P* < 0.001 are significantly different from the IMQ group. Here PL = PPL 10 mg/kg and PH = PPL 30 mg/kg topically, PSC = PPL 1 mg/kg subcutaneously, and TAC = Tacrolimus 20 mg/kg topically.

IMQ topical application induces local and systemic inflammation, which is accompanied by altered epidermal integrity and splenomegaly^22^. To assess the effect of PPL treatment on these phenotypic changes, we applied IMQ cream on the shaved dorsal skin of mice daily for 6 days. Initially, a pilot study was performed to determine the optimal dose of PPL. Based on the results observed from the pilot study, 10 (PL = PPL low dose) and 30 (PH = PPL high dose) mg/kg dose of PPL was fixed for topical intervention, which was incorporated in the 0.75% cabapol gel after optimization based on shear stress vs shear rate (Fig. S1a) and viscosity vs shear rate (Fig. S1b), while a dose 1 mg/kg (PSC = PPL subcutaneous) of PPL treatment was fixed for subcutaneous administration. For skin compliance and safety evaluation of PPL alone in topical and subcutaneous routes, an animal study was carried out in BALB/c mice, where PPL has not shown significant clinical signs of toxicity and the detailed method is described in Supplementary Data (Figs. S2a–f and S3a–f). Marketed 20 mg/kg of TAC ointment was used as a standard to compare the effects of PPL; this cream was applied directly via topical route without gel as the main base for the ointment was carbopol. From the third day of IMQ application, PPL has been administered every day at various doses along with TAC as mentioned above for 4 consecutive days. IMQ topical application profoundly increased erythema, scaling, and thickening of the skin and left ear after day 6 compared with the control group animals, whereas PH- and PSC-treated groups showed a notable decrease in the phenotypic changes and severity index scoring (Fig. 1a, c–e). IMQ application induces splenomegaly through systemic inflammatory effects, which is the another characteristic feature of mice model, which might appear due to increased immunological activity and hyperplasia^23,24^. In this context, we found that PPL treatment significantly attenuated the enlarged spleens (Fig. 1b), and the spleens from all the animals in every group were quantified based on the spleen weight/body weight index (Fig. S4a). As shown in Fig. 1f and Fig. S4b, the skin folds and ear thickness differences were visible in the IMQ group from day 2 and gradually increased throughout the study. Similarly, hematoxylin and eosin (H & E; Fig. 1g and Fig. S4c), as well as 4′,6-diamidino-2-phenylindole (DAPI) staining (Fig. 1h and Fig. S4d), of mouse back skin showed that IMQ application induces parakeratosis and epidermal acanthosis. Moreover, the epidermal thickness as well as ear thickness was reduced significantly in the PPL-treated mice groups. We next evaluated the effect of PPL on the IMQ-induced expression of the Akt pathway and proliferative markers. It was observed that IMQ increases the phosphorylation of Akt at both Ser473 and Thr308 sites, as well as downstream p70S6K (Thr389). Furthermore, a marked increase in the expression levels of mammalian target of rapamycin (mTOR), Ki67, and Cyclin D1 has been observed. However, no significant effect was observed toward proliferating cell nuclear antigen (PCNA) and Bcl-2 protein expression. The topical intervention of PPL in the PH and PSC groups significantly reduced the phosphorylation of Akt at Ser473, whereas at Thr308 site, phosphorylation was reduced significantly in the PSC group but not topically. However, p70S6K (Thr389) phosphorylation was significantly inhibited only in the PH group treatment. Moreover, the proliferative markers Ki67, PCNA, and Cyclin D1 expression levels were reduced along with anti-apoptotic Bcl-2 protein (Fig. 1i and Fig. S4e–l). Collectively, these results show that PPL reduces epidermal hyperproliferation by targeting Akt signaling and by inhibiting proliferative markers.

### Inhibitory effects of PPL on keratinocyte hyperproliferation by inducing apoptosis

**Fig. 2:**
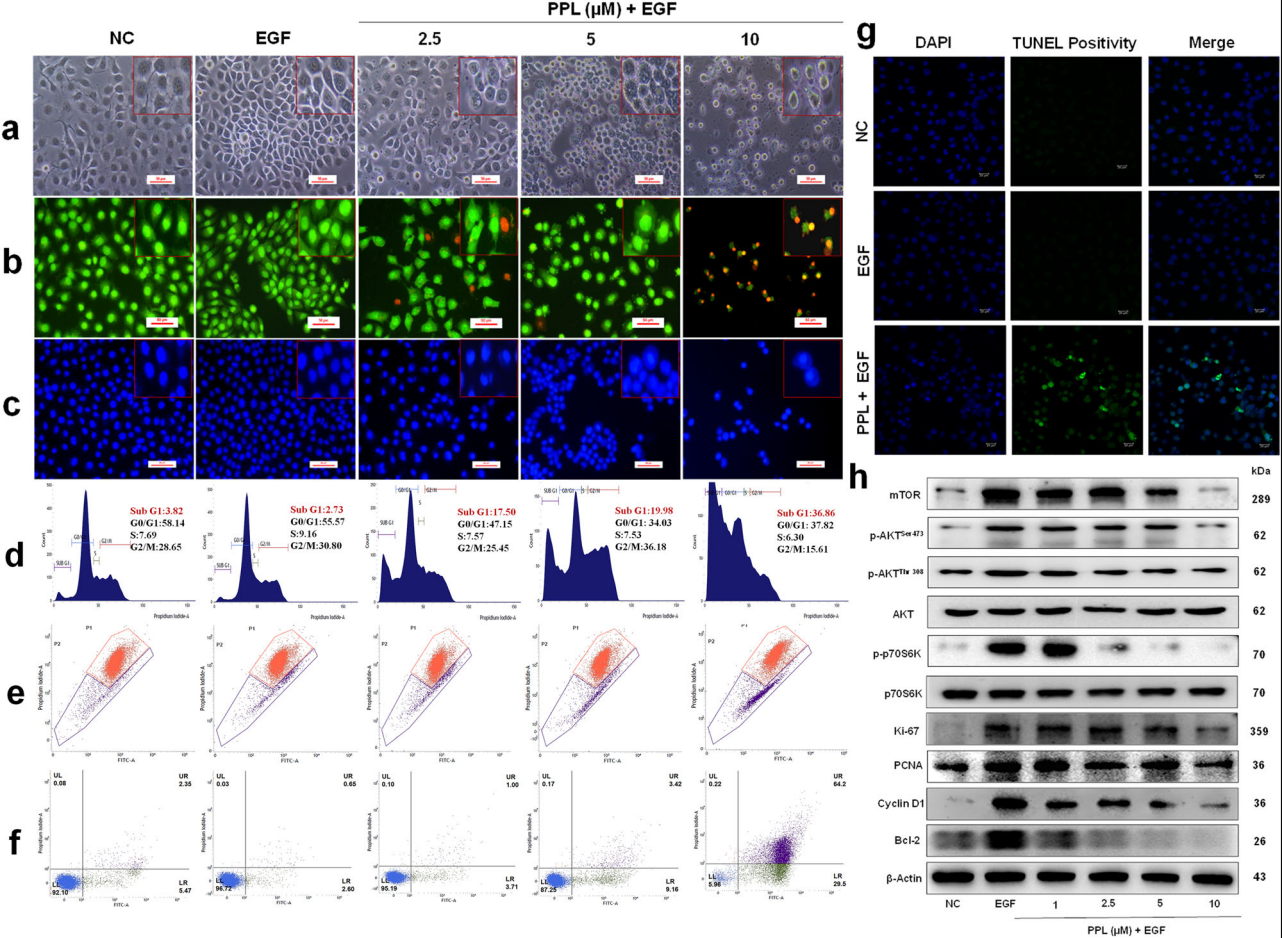
PPL inhibits keratinocytes hyperproliferation by induction of apoptosis. HaCaT cells were pretreated with PPL at the indicated concentrations; after 2 h, EGF (50 ng/ml) was stimulated for 48 h and assessed for **a** morphological changes induced by PPL under phase contrast microscope. **b** AO/EB dual staining and **c** DAPI staining were performed to visualize the apoptotic and nuclear changes by using fluorescent microscope at ×200 magnifications. Flow cytometric analysis was performed to determine the **d** distribution of PI labeling in different phases. The peaks in the histograms correspond to Sub G1, G0/G1, S, and G2/M phases of the cell cycle. **e** JC-1 staining was performed to analyze the loss of mitochondrial membrane potential (ΔΨm) by PPL. P1 represents the formation of J-aggregates in healthy mitochondria, whereas P2 represents the loss of ΔΨm in cells due to the presence of J-monomers. **f** Apoptosis in cells was measured by staining with Alexa Flour 488 Annexin V and PI. The percentage of cells positive for Annexin V-Alexa Flour 488 and/or PI in the quadrants were quantified. Cells in the upper left quadrant (Q1-UL; AV−/PI+): necrotic cells; lower left quadrant (Q2-LL; AV−/PI−): live cells; lower right quadrant (Q3-LR; AV+/PI−): early apoptotic cells and upper right quadrant (Q4-UR; AV+/PI+): late apoptotic cells. **g** Increase in the TUNEL positivity as an indicative of apoptotic DNA fragmentation with PPL was measured by TUNEL staining after 48 h incubation and images were captured at ×400 magnification. **h** For checking the phosphorylation of Akt signaling, HaCaT cells were pretreated with PPL for 12 h and stimulated the cells with EGF (50 ng/ml) for 30 min, whereas mTOR, Ki67, PCNA, Cyclin D1, and Bcl-2 expression was determined by pretreating the cells with PPL for 2 h and stimulated the cells for 12 h with EGF. Later, whole-cell extract was subjected to western blotting. PPL counteracts EGF-induced proliferation and apoptosis marker protein expression in HaCaT cells. β-Actin or respective totals were used as an endogenous loading controls.

The persistent keratinocyte proliferation and evasion of apoptosis signaling are the hallmarks of psoriasis^25^. EGF regulates keratinocyte growth, proliferation, and differentiation by binding to epidermal growth factor receptor (EGFR) and maintain high proliferative state^26,27^. Apoptosis has been proposed as a mechanism that disrupts abnormal epidermal thickness in psoriasis^28^. To investigate the role of PPL on EGF-induced hyperproliferation, 3-(4,5-dimethylthiazol-2-yl)-2,5-diphenyltetrazolium bromide (MTT) assay was performed in the presence of EGF, which showed that PPL effectively reduced the EGF-stimulated cell viability of keratinocytes at both 24 (Fig. S4a) and 48 h (Fig. S4b) in a concentration-dependent manner, respectively. On the other hand, PPL alone effect was analyzed at 96 h, where there was no significant change in the cell viability up to 6.25 μM concentration, and PPL alone was found to be less effective in comparison with EGF-stimulated conditions (Fig. S9a). Further, cells were morphologically examined after PPL treatment, where apoptotic cells were stained with Acridine orange (AO)/Ethidium bromide (EB) and DAPI. Here we found that cells have lost their morphology and attained spherical shape with prominent apoptotic blebs and chromatin condensation at 5 and 10 µM concentrations (Fig. 2a–c). Furthermore, the effect on the percentage of cell cycle phase distribution was analyzed by flow cytometry. As shown in Fig. 2d and Fig. S5c, PPL treatment increased the proportion of Sub-G1 phase population significantly at 5 and 10 µM compared to untreated controls, which is an indicator of apoptosis as a result of inter-nucleosomal DNA fragmentation, which was further confirmed by terminal deoxynucleotidyl transferase dUTP nick-end labeling (TUNEL) assay (Fig. 2g and Fig. S5n). Moreover, 5,5′,6,6′-tetrachloro-1,1′,3,3′-tetraethylbenzimidazolocarbocyanine iodide (JC-1) staining and Annexin V-binding assay showed that PPL treatment induced the loss of mitochondrial membrane potential (∆Ψm) and exhibited moderate early and significant late apoptosis at higher concentrations (Fig. 2e, f and Fig. S5d, e). Under the EGF stimulation and/or along with oxidative stress conditions in the presence of 300 μM H_2_O_2_ conditions, 2',7'-dichlorodihydrofluorescein diacetate (DCFDA) staining was performed, where PPL at 5 and 10 µM concentration increased the reactive oxygen species (ROS) levels and a marked increase in oxidative stress were observed when combined with H_2_O_2_ (Fig. S5o). Akt/mTOR pathway activates Ki67, Cyclin D1, and PCNA proliferative markers, which play a pivotal role in mediating hyperproliferation^29^. PPL treatment significantly reduced the phosphorylation of Akt and p70S6K. On the other hand, a significant reduction in Cyclin D1, Bcl-2, PCNA, and Ki67 protein expression levels were observed (Fig. 2h and Fig. S5f–m). These results clearly demonstrate that PPL induces apoptosis and inhibits keratinocyte hyperproliferation.

### PPL suppresses the expression of STAT3 and K17 in HaCaT cells and skin tissues

**Fig. 3:**
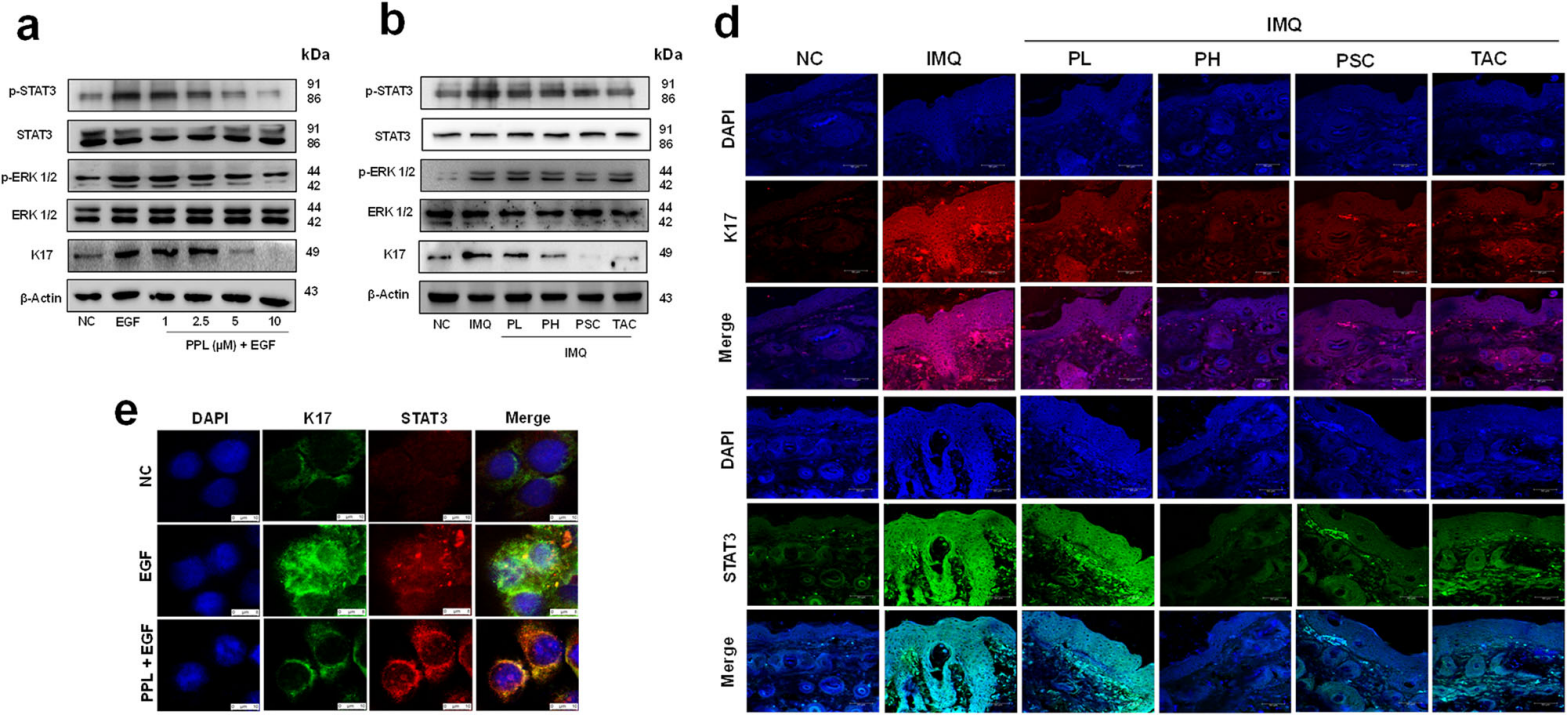
PPL suppresses keratin 17 (K17) by inhibiting STAT3 phosphorylation. **a** HaCaT cells were pretreated with PPL for 12 h and stimulated with EGF (50 ng/ml) for 1 h. The protein was isolated and the samples were subjected to western blot analysis to determine the expression of p-STAT3, p-ERK 1/2, and K17 in **a** HaCaT cells and **b** skin tissue lysates. **c** Immunofluorescence was performed to analyze the expression of STAT3 and K17 in keratinocytes. **d** IF analysis shows attenuation of STAT3 and K17 expression in skin tissue sections with PPL treatment. Here PL = PPL 10 mg/kg and PH = PPL 30 mg/kg topically, PSC = PPL 1 mg/kg subcutaneously, and TAC = Tacrolimus 20 mg/kg topically.

The mounting evidence suggest that K17 is involved in cell proliferation and differentiation, which is expressed aberrantly in the suprabasal keratinocytes of psoriatic lesions, primarily regulated by the cytokines through STAT3 and ERK1/2 signaling^8,30^. In the present study, we found that EGF (50 ng/ml) stimulation for 60 min effectively upregulated the expression of K17, which might be due to the increased phosphorylation of STAT3 at Tyr705 and ERK1/2. While the treatment with PPL remarkably suppressed the expression of K17 by inhibiting STAT3 phosphorylation; however, no significant effect was found toward the phosphorylation of ERK1/2 (Fig. 3a and Fig. 6a–c). Moreover, we have observed that PPL alone had reduced the phosphorylation of STAT3 in HaCaT cells, which was observed at 96 h (Fig. S9b, c). We further examined consistent findings in vivo, where K17 expression and the phosphorylation of STAT3 were inhibited significantly in either route of PPL treatment (Fig. 3b and Fig. S6d–f). Thereafter, immunofluorescence (IF) analysis was performed in HaCaT cells (Fig. 3c) and tissues (Fig. 3d and Fig. S6g, h), where with EGF and IMQ treatment the STAT3 and K17 positivity has been increased as compared to control, whereas with PPL treatment a significant decrease in the immunopositivity of STAT3 and K17 was observed. This provides an evidence that PPL effectively suppress the STAT3 signaling, thereby inhibiting K17.

### PPL inhibits pro-inflammatory cytokine and chemokine levels in IMQ-induced psoriasis

**Fig. 4:**
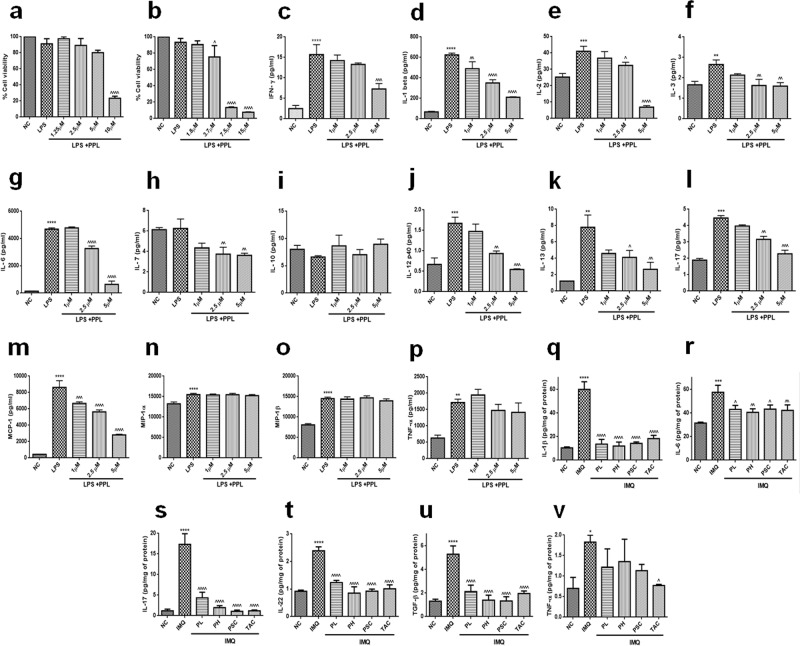
PPL suppresses inflammatory stimuli-mediated cytokines and chemokine levels. MTT assay was performed to determine the cell viability of RAW 264.7 cells by stimulation with LPS (1 μg/ml) after pretreatment of PPL for 2 h and incubated for **a** 24 and **b** 48 h. **c** Cytokine and chemokine profiling was performed in murine macrophages, where cells were pretreated with 0.5, 1, and 2.5 μM concentrations of PPL for 2 h and then stimulated with 1 μg/ml of LPS and incubated for another 24 h and the levels of **c**–**l** cytokines and **m**–**o** chemokines were determined by multiplex analysis. **p**–**v** In addition, PPL effectively ameliorates inflammatory cytokine production in skin tissues. The data represent mean ± SD (in vitro, *n* = 3; in vivo, *n* = 5 mice per group). *P < 0.05, **P < 0.01, ***P < 0.001, and ****P < 0.0001 vs. NC group. ^*P* < 0.05, ^^*P* < 0.01, ^^^*P* < 0.001, and ^^^^*P* < 0.0001 are significantly different from the LPS/IMQ group. Here PL = PPL 10 mg/kg and PH = PPL 30 mg/kg topically, PSC = PPL 1 mg/kg subcutaneously, and TAC = Tacrolimus 20 mg/kg topically.

The pro-inflammatory cytokines and chemokines gets activated aberrantly, thereby involved in the recruitment of inflammatory cells to keratinocytes, which leads to skin inflammation. To test the effect of PPL on the levels of these cytokines and chemokines, initially, we determined the effect of PPL on the viability of RAW 264.7 cells, where pretreatment was given with PPL at twofold serial dilutions, after 2 h cells were stimulated with lipopolysaccharide (LPS). It was found that PPL has not shown discernible toxicity up to 5 µM concentrations at 24 h (Fig. 4a), while an increase in the cell death was observed from 3.7 µM concentrations at 48 h time point (Fig. 4b). Moreover, we have tested the effect of PPL alone without LPS stimulation up to 96 h, where PPL was found to be minimally toxic up to 5 µM and a significant reduction in the cell viability was observed from 6.25 µM concentration (Fig. S10a). Hence, 1, 2.5, and 5 µM concentrations of PPL were pretreated for 2 h, followed by induction of inflammation with LPS (1 μg/ml) and incubated further for 24 h. It was observed that LPS induced pro-inflammatory cytokines/chemokines, including IFN-γ, IL-1β, IL-2, IL-3, IL-6, IL-7, IL-12 p40, IL-13, IL-17, TNF-α, monocyte chemoattractant protein-1 (MCP-1), and macrophage inflammatory protein (MIP)-1α and -1β, whereas reduced levels of anti-inflammatory cytokine IL-10 was observed. PPL treatment significantly inhibited these cytokines but no significant effect was found toward IL-10, TNF-α, and chemokines MIP-1α along with MIP-1β (Fig. 4c–p). Topical IMQ prominently induced the levels of pro-inflammatory cytokines, including IL-1β, IL-6, TNF-α, IL-17, IL-22, and transforming growth factor (TGF)-β, while PPL significantly suppressed these levels (Fig. 4q–v). Therefore PPL has shown profound anti-inflammatory effects through the abrogation of cytokine and chemokine network.

### PPL abrogates the inflammation regulated protein expression and epigenetically regulates histone-modifying enzymes in macrophages upon LPS exposure

**Fig. 5:**
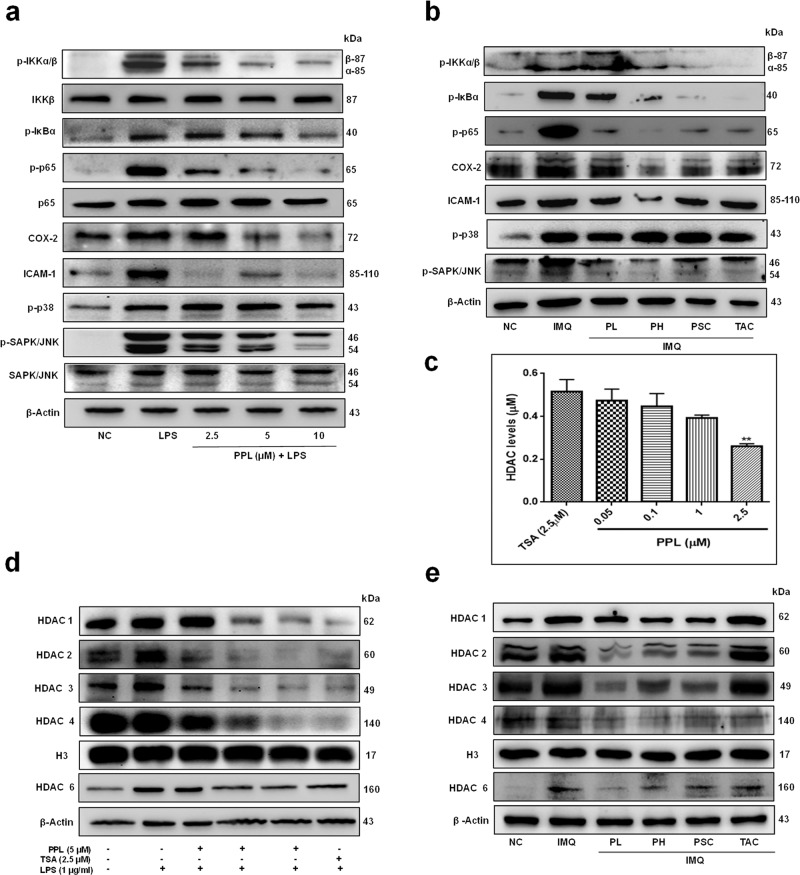
PPL inhibits the protein expression of the inflammatory signaling cascade and exhibits potent HDAC inhibitory activity in murine macrophages and skin tissues. PPL potently inhibits LPS- and IMQ-induced IKK-mediated downstream signaling but no modulation in the MAP kinase pathways. **a** RAW 264.7 cells were pretreated with PPL for 12 h and stimulated with LPS for 30 min, and western blot analysis was performed with the indicated antibodies. **b** Immunoblots of p65 and downstream signaling markers with PPL treatment observed in skin tissues. **c** HDAC inhibitory activity of PPL was analyzed by HDAC fluorometric kit at 0.05, 0.1, 1, and 2.5 µM concentrations of PPL, and HDAC inhibitory activity was compared with trichostatin A (TSA) at 2.5 µM concentrations. **d** RAW 264.7 cells were pretreated with PPL at the indicated concentrations for 2 h, and later cells were stimulated with LPS (1 µg/ml) and incubated for 12 h. Nuclear lysate was extracted and analyzed for the preferential overexpression of HDAC1–4 with LPS, abrogation in the expression by PPL treatment were analyzed by western blotting and compared with H3 expression, and HDAC6 expression was analyzed in whole-cell extract and compared with β-Actin expression. **e** Similarly, PPL epigenetic regulation by reducing HDAC1–4 expression were analyzed in psoriatic and PPL-treated skin tissue nuclear extracts; HDAC6 were analyzed in the whole-cell extracts of skin tissue. The data represent mean ± SD (*n* = 3). **P < 0.01 is significantly different from the TSA. Here PL = PPL 10 mg/kg and PH = PPL 30 mg/kg topically, PSC = PPL 1 mg/kg subcutaneously, and TAC = Tacrolimus 20 mg/kg topically.

Macrophages are crucial in mediating the psoriatic inflammation and MCP-1 has strong macrophage-recruiting properties^31^. Wang et al. have reported that depleting macrophages results in reduced inflammation and significant improvement in psoriasis-associated symptoms^32^. From the previous reports, it was also found that macrophages were activated aberrantly with IMQ topical application^33^. On the other hand, p65 is a key transcription factor that orchestrates inflammation by expressing a large number of pro-inflammatory genes^34^. In the line with these evidences, and to investigate this mechanism, we treated RAW 264.7 cells with LPS, which elicited a marked IκBα phosphorylation mediated by IκB kinase (IKK α/β complex), and this led to an increase in the p65 phosphorylation, which further activated the expression of COX-2. PPL treatment resulted in inhibition of IKK α/β and IκB phosphorylation with a strong reduction in the phosphorylation of p65. p65 and STAT3 cooperatively recruit intercellular adhesion molecule (ICAM)-1^35^, which play an important role in inflammatory cell migration in psoriasis; moreover, PPL reduced LPS-induced ICAM-1 expression. In addition, PPL moderately reduced the p-p65 expression even in the absence of LPS, which was observed at 96 h (Fig. S10b, c). On the other side, MAPKs including p-p38 and p-JNK were found to be elevated upon LPS stimulation. It was found that, with PPL intervention, p-p38 MAPK expression remains unchanged with a significant decrease in JNK phosphorylation (Fig. 5a and Fig. S7a–g) and these results were consistent when observed in vivo (Fig. 5b and Fig. S7h–n).

Previous studies demonstrated that epigenetic modifications by HDAC inhibition exhibits potent anti-inflammatory activity by inhibiting cytokine synthesis along with p65 and STAT3^10,13,36–39^. This frame of reference pondered our interest to explore the effects of PPL in modulating epigenetics. Initially, to analyze the effect of PPL on HDACs, we used a fluorometric HDAC assay kit. Here trichostatin served as a standard to measure the activity. HDAC inhibitory activity of PPL was tested on the HeLa cell lysate at four different concentrations (0.05, 0.1, 1, and 2.5 µM) along with the standard trichostatin (trichostatin A (TSA); 2.5 µM). The results showed that HDAC levels were reduced significantly with 0.44 ± 0.06, 0.49 ± 0.13, 0.26 ± 0.03, and 0.24 ± 0.01 µM, respectively, at 0.05, 0.1, 1, and 2.5 µM concentrations of PPL. These results were compared with TSA, where HDAC levels were found to be 0.52 ± 0.02 µM (Fig. 5c). Subsequently, it was observed that LPS regulated the HDACs at the protein level such as HDAC1, 2, 3, and 6. Moreover, PPL treatment effectively reduced HDAC expression and the results were compared with TSA observed in macrophages (Fig. 5d and Fig. S7o–s). Similar observations were found with IMQ induction, where HDAC1, 3, and 6 expression levels were found to be elevated significantly and an effective reduction was found with PPL at the protein level (Fig. 5e and Fig. S7t–x). These results demonstrate that PPL effectively inhibits inflammatory NF-κB signaling and epigenetically modulates HDAC expression.

### PPL inhibits IκBα-mediated p65 and HDAC3 nuclear translocation

**Fig. 6:**
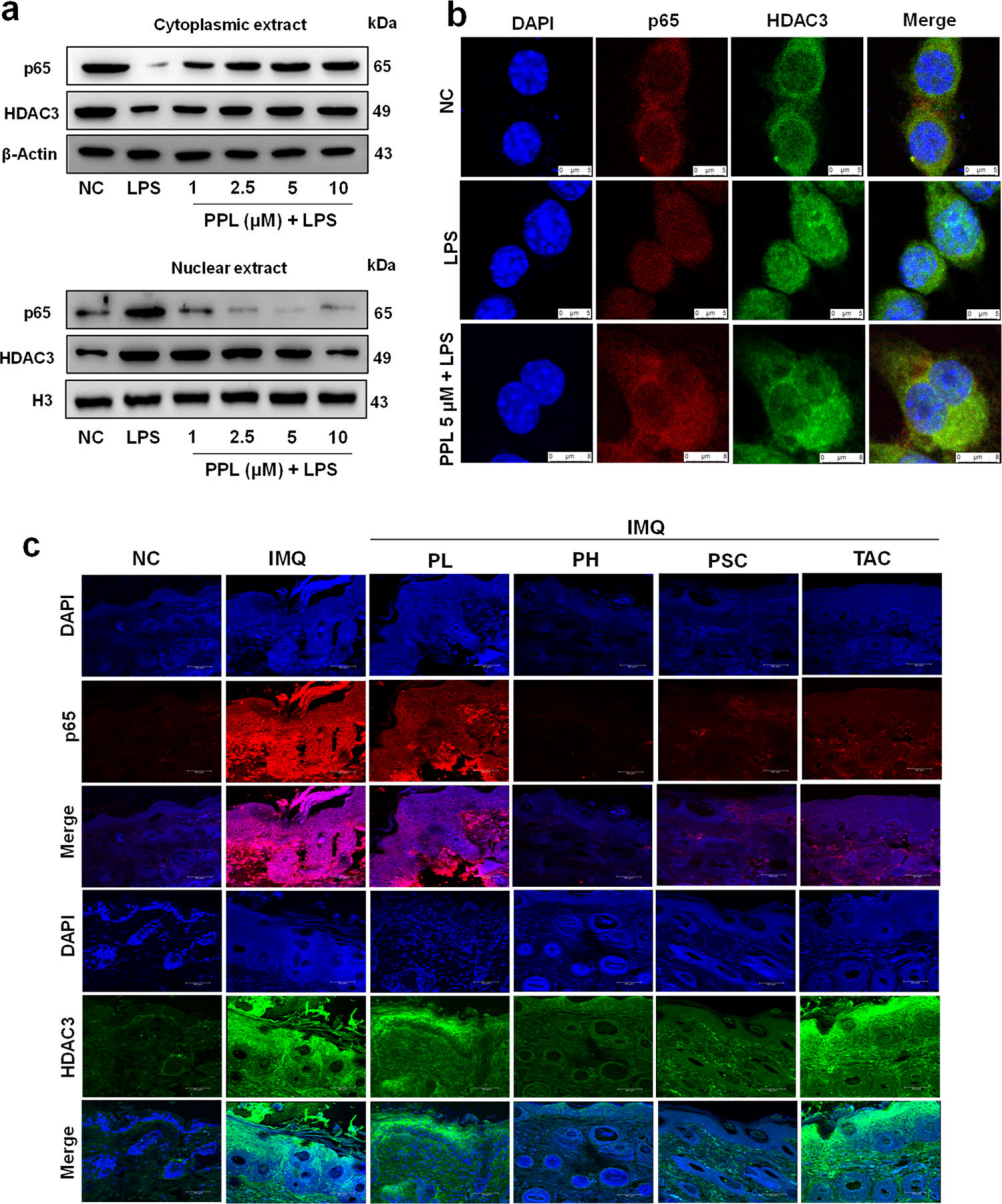
PPL potently inhibits the nuclear translocation of p65 and HDAC3 induced by LPS. **a** RAW 264.7 cells were grown in full medium and then serum starved for 6 h with 0.1% FBS and treated with PPL at different concentrations indicated; after 12 h, cells were stimulated with LPS (1 µg/ml) and further incubated for 30 min. p65 and HDAC3 expression was analyzed in both cytoplasmic and nuclear fractions, where β-Actin and H3 were the loading controls, respectively. **b** Immunocytochemical analysis was performed to determine the cellular distribution of p65 and HDAC3 in RAW 264.7 cells and stained with p65 antibody (red), HDAC3 antibody (green), and DAPI for nuclear staining (blue) following treatment of the cells with LPS (1 μg/ml) and PPL (5 µM) + LPS (1 μg/ml). Images were captured at ×630 magnification. **c** IF results showed that PPL downregulated the expression of p65 (Red) and HDAC3 (Green) in the skin tissue sections. Here PL = PPL 10 mg/kg and PH = PPL 30 mg/kg topically, PSC = PPL 1 mg/kg subcutaneously, and TAC = Tacrolimus 20 mg/kg topically.

Under basal conditions, HDAC3 protein is associated with IκBα in the cytoplasm. With inflammatory stimuli, HDAC3 significantly translocates into the nucleus^40^. Similarly, p65 also forms the complex with IκBα and initiates gene transcription when translocated into the nucleus^11^. The compounds which hampers the nuclear translocation interferes with the inflammatory gene transcription, which further reduces inflammation. To investigate this mechanism, cells were stimulated with LPS and the expression of HDAC3 and p65 were evaluated in the cytoplasm and nuclear extracts. Western blotting results indicated that PPL treatment significantly inhibited the nuclear translocation of both HDAC3 and p65 concomitantly through the inhibition of IκBα phosphorylation (Fig. 6a and Fig. S8a–d). Subsequently, we examined confocal analysis and potent inhibition of HDAC3 and p65 expression has been observed with PPL treatment upon LPS stimulation in macrophages (Fig. 6b). In addition, we have examined the expression of p65 and HDAC3 in skin tissue sections by IF analysis, which revealed a dramatic reduction in the expression of both proteins in topical and SC routes (Fig. 6c and Fig. S8e, f). These results suggest that one of the prominent effects of PPL in reducing inflammation is through the effective inhibition of nuclear translocation of p65 and HDAC3 and thereby inhibits inflammatory gene transcription.

### PPL augments the p65:IκBα:HDAC3 complex formation in the cytoplasm by enhancing protein–protein interaction with IκBα

**Fig. 7:**
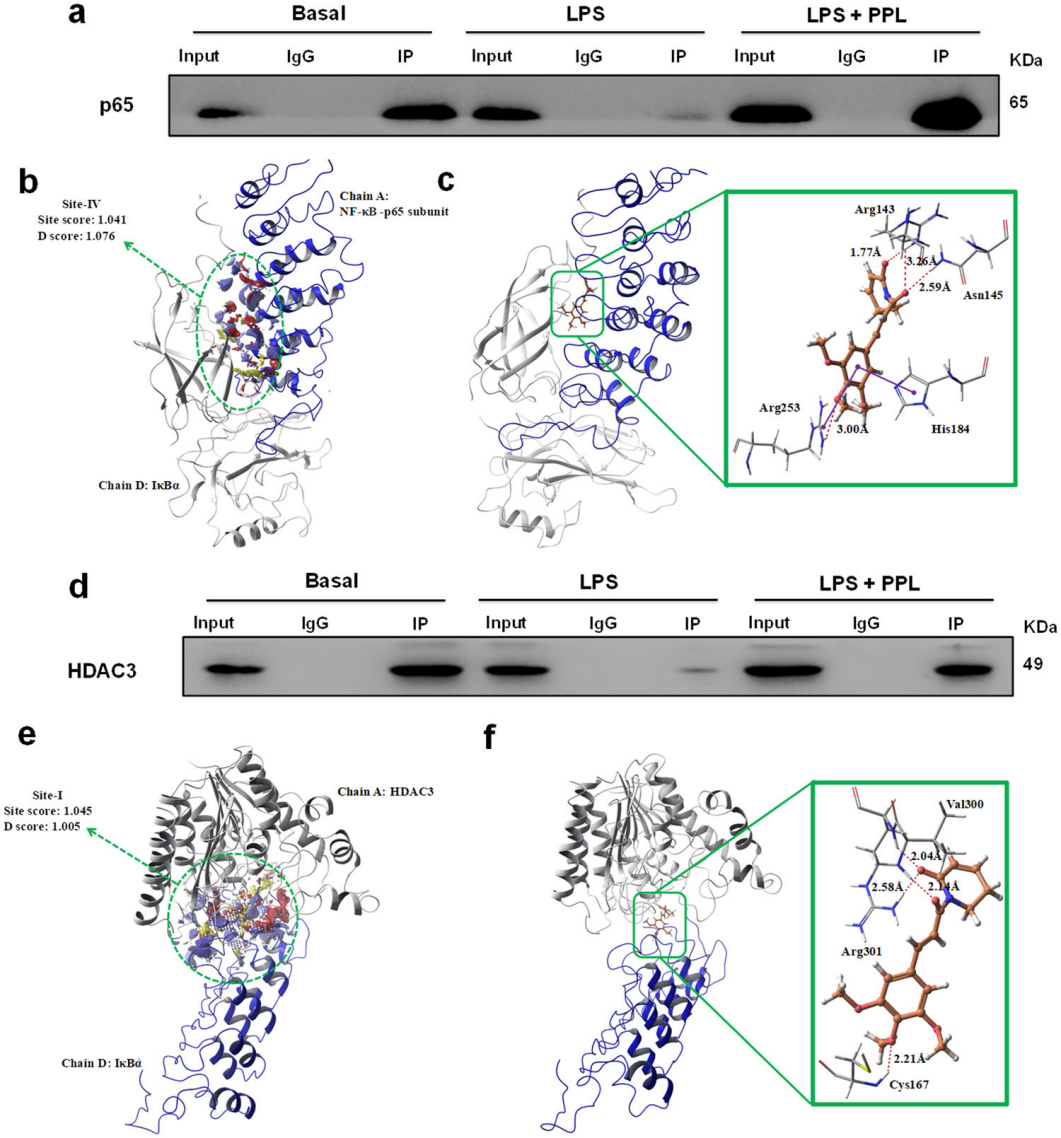
PPL enhances IκBα-HDAC3-p65 complex. **a** Co-immunoprecipitation (Co-IP) was performed in the cytoplasmic extract from RAW 264.7 cells to determine the association of IκBα-p65 in basal condition, upon LPS stimulation and with PPL treatment. IgG was used as a control for nonspecific signal. **b** Site points (white spheres) and SiteMap surface (red and blue color contours) for Site-IV-binding site of p65/IκBα protein complex. **c** Docking model of PPL in binding site (Site-IV) of p65/IκBα protein complex and its ligand–protein interactions. The dark red dashed lines represent hydrogen bonds. H-bond distances (in Å) between hetero atoms of ligand and amino acid residues are as follows: Arg143 (1.77 and 3.26), Asn145 (2.59), and Arg253 (3.00). The blue line indicates arene–arene interaction with His184 and Arg253. **d** Co-IP was performed in the cytoplasmic extract from RAW 264.7 cells to determine the association of IκBα-HDAC3 in basal condition, upon LPS stimulation and with PPL treatment. **e** Site points (white spheres) and SiteMap surface (red and blue color contours) for Site-I-binding site of HDAC3/IκBα protein complex. **f** Docking model of PPL in binding site (Site-I) of HDAC3/IκBα protein complex and its ligand–protein interactions. The dark red dashed lines represent hydrogen bonds. H-bond distances (in Å) between hetero atoms of ligand and amino acid residues are as follows: Cys167 (2.21), Val300 (2.14), and Arg301 (2.04 and 2.58).

Protein–protein interactions play a key role in a variety of diseases. Molecular docking could be a helpful tool to develop the potential binding sites of drug candidates that target the protein–protein interfaces^41^. P65:IκBα:HDAC3 interact and remain as a complex mainly in the cytoplasm of unstimulated cells. Upon stimulation by pro-inflammatory cytokines, viral infections, or pathogens, IκBα gets phosphorylated and degraded such that p65 and HDAC3 remains free in the cytoplasm, which further translocates into the nucleus. The association of this interaction and complex formation in the cytoplasm is a prerequisite in inhibiting various gene expression and is of obvious clinical significance^40,42^. In the line of these evidences, we first demonstrated the protein–protein interactions and binding of PPL by molecular modeling. The results from Tables S1–S3 demonstrate the molecular docking along with H-bonding as well as hydrophobic and arene–arene interactions of PPL with p65/IκBα (Fig. 7b, c) and HDAC3/IκBα protein complexes (Fig. 7e, f).

We next investigated the association of HDAC3 and p65 with IκBα experimentally by using co-immunoprecipitation (Co-IP) in the cytoplasmic extract. We found that in the basal conditions HDAC3 and p65 were found in the IP product of IκBα antibody, while with LPS stimulation for 30 min potently disrupted the coexisting protein–protein interaction of HDAC3 and p65 with IκB-α, which was evident from the IP fraction of LPS stimulation. The association of HDAC3 and p65 with IκBα was investigated with PPL treatment. It was indicated from the IP fraction that PPL treatment potently abolished the LPS-induced dissociation of p65 and HDAC3. By confirming the above outcomes, it was observed that the amplitude of HDAC3 and p65 nuclear/cytosol oscillations in mediating inflammation was reduced by PPL through the inhibition of IκBα phosphorylation, which may have significance in attenuating inflammatory gene expression (Fig. 7a, d).

## Discussion

The proliferation and differentiation of keratinocytes are stimulated by various growth factors and cytokines^43,44^ with substantial acceleration in cell cycle time, which shortens from 311 h in normal to 36 h in psoriatic lesions^45^ and possess enhanced ability to resist apoptosis^46,47^. EGF is one of the growth factors that stimulates proliferation and differentiation by binding with the EGFR and regulates cell growth and division in psoriasis^26,48–50^. Although traditional treatment interventions are effective, lacunae in the therapies exist due to the adverse effects and inconvenience, since psoriasis is a chronic disease that warrants safe and effective therapies long term^51,52^. The miracle properties of PPL such as potent anti-proliferative and anti-inflammatory characteristics prompted us to explore the potential in psoriasis. Our findings strongly demonstrate that PPL alleviates psoriasis by abrogating hyperproliferation and inflammation in keratinocytes and macrophages, respectively.

**Fig. 8:**
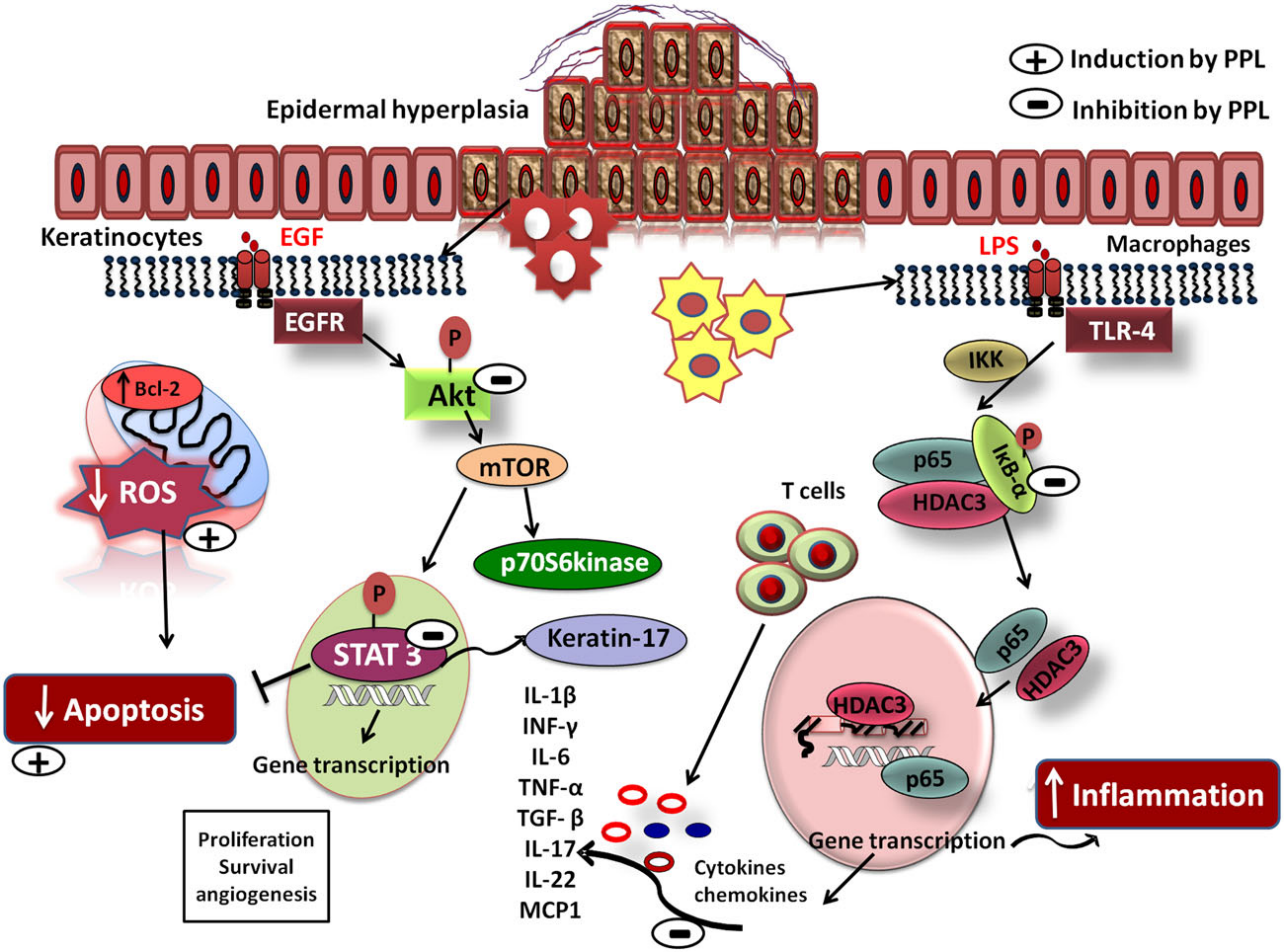
PPL ameliorates psoriatic-like skin inflammation by inhibiting hyperproliferation and inflammation. Schematic illustration of the effect of PPL on Akt/mTOR/STAT3 cascade and apoptosis. PPL inhibits upstream and downstream signaling in keratinocytes resulting in decreased cell proliferation and induction of apoptosis and promotes differentiation by inhibiting keratin17. Simultaneously abrogation of HDAC3 and p65 nuclear translocation was observed in murine macrophages by inducing association of HDAC3 and p65 to IκBα, thus reducing inflammation and cytokine/chemokine levels and also normalizing the tissue architecture.

Initially, we determined the cell viability assay with EGF stimulation in keratinocytes, where inhibition of cell viability was observed from 3.75 μM at 48 h time point. We next evaluated the cell viability without EGF and incubated further for 96 h, where reduction in the cell viability was observed from 6.25 μM. From a mechanistic point of view, we sought to investigate the effect on apoptosis signaling. Based on fluorescence staining, AO/EB, and DAPI, it was found that PPL prominently induced apoptosis with shrinkage of cytoplasm, nuclear fragmentation, and nuclear pyknosis at 5 and 10 µM concentrations. The significant late apoptosis and modest early apoptotic cell death with negligible necrosis were evident from Annexin V/propidium iodide (PI) assay in the treated groups. Furthermore, a notable Sub-G1 phase cell cycle arrest was observed as a result of DNA fragmentation in apoptosis, which was further confirmed by TUNEL assay. Increased expression of antioxidant regulator Nrf2 was found to enhance cell proliferation in psoriasis^53^, while another study demonstrated the importance of hyperbaric oxygen therapy, which elevates tissue ROS levels that is effective in attenuating hyperproliferation^54^. To test this hypothesis, DCFDA and JC-1 analysis was performed, where PPL intervention led to an increase in the ROS generation in EGF-stimulated cells through the dissipation of ΔΨm. Interestingly, our in vivo results evidenced that PL, PH, and PSC treatment groups exhibited reduced severity index with decreased skin fold and ear thickness as compared to the IMQ group and a strong reduction in the splenomegaly was observed, which was induced by IMQ through systemic effects. Consistent with in vitro results, a sharp decrease in the epidermal hyperproliferation was observed along with reduced parakeratosis, which were evident from H & E along with DAPI staining.

We next investigated the skin compliance of PPL on normal skin, where PPL was applied topically and via SC route as a daily regimen for 7 and 21 days and monitored every day for the PPL-induced phenotypic changes. We found no significant changes such as erythema, itchiness, acute inflammatory responses, and body weight reduction. The histopathological evaluation shows that PPL minimally reduced the epidermal thickness at both time intervals, but this change was found to be insignificant. Furthermore, we have analyzed the effects of PPL on p-p65 and p-STAT3 expression at both time intervals from skin tissue samples. However, in either route of administration PPL did not show significant effect on the phosphorylation of both proteins. It is clear from these data that PPL treatment has acceptable skin compliance and did not show any type of inflammatory response. Molecularly, PPL intervention dampened the expression of cell growth promoters and proliferation markers, including p-Akt at Ser473 and Thr308 sites, p70S6 kinase, mTOR, Ki-67, and Cyclin D1, and reduced the anti-apoptotic BCL2 expression both in vitro and in vivo.

K17 expression expressed aberrantly in the suprabasal keratinocytes, which is regarded as a hallmark of psoriasis that is primarily induced by IL-17A through STAT3 and ERK1/2, thus promoting the proliferation of keratinocytes and T cells^8,30,53^. Our findings indicate that EGF in keratinocytes and IMQ in skin tissues effectively enhanced the phosphorylation of STAT3 and ERK1/2, thus activating the K17. PPL treatment effectively abrogated the STAT3 and K17, while no significant change in the ERK1/2 expression was observed.

The serum levels of cytokines, which includes TNF-α, IFN-γ, IL-2, IL-6, IL-8, IL-18, IL-22, and IL-23, are the potential biomarkers for psoriasis^55^, which are primarily produced by Th1 and Th17 cells in coordination with dermal dendritic cells, macrophages, mast cells, and neutrophils^56^. The previous study by Xiao et al. has reported the potential of PPL in alleviating inflammatory cytokines induced by LPS in dendritic cells and reduction of alloproliferation of T cells^57^. Here we have studied the effect of PPL on LPS-stimulated macrophages, which induced the array of cytokines and chemokines where a remarkable reduction in the level of pro inflammatory cytokines and also a chemokine MCP-1 was observed, while the levels of other chemokines such as MIP-1α and MIP-1β remain unchanged.

Aberrant epigenetic regulation leads to inflammation in psoriasis^58,59^. Previous studies have shown that HDAC1 is upregulated in the biopsies of psoriatic lesions^60^. HDACs inhibition has been found to exhibit potent anti-inflammatory effects by inhibiting macrophage activation in inflammatory disorders^36,61^. From a mechanistic point of view, increasing evidence has suggested that HDAC3-deficient macrophages upon LPS stimulation could not activate almost half of the inflammatory gene expression^62^. For instance, it was reported that HDAC inhibition by TSA treatment and vorinostat impedes the conversion of regulatory T cells (Tregs) into IL-17^+^ Tregs^12,63^. This led us to hypothesize the effect of PPL on modulating HDAC expression. Interestingly, PPL intervention strikingly inhibited the HDAC enzyme activity and reduced the HDAC expression in macrophages and skin tissues and further attenuated the p65 signaling cascade including ICAM-1 expression.

In resting cells, approximately 100,000 p65 dimers are bound to IκBα along with HDAC3, which are sequestered in the cytoplasm; with inflammatory stimulation, IκBα gets degraded and HDAC3 and p65 are translocated to the nucleus and are involved in inflammatory-regulated gene expression^11,40,64^. It has been found in this present study that LPS stimulation profoundly induced the nuclear translocation of these proteins, whereas PPL treatment significantly hampered the relocation of these proteins to the nucleus, which was evidenced in macrophages from immunoblotting and confocal analysis, and consistent result has been found in the skin tissues where reduced expression has been observed.

Our study next focused to delineate the mechanism of PPL in mediating the interaction of p65 and HDAC3 to IκBα in the cytoplasm, which was determined by using Co-IP in the cytoplasmic extract and molecular docking. Co-IP results showed HDAC3 and p65 were strongly bound to IκBα in basal conditions. Next, we confirmed with the LPS stimulation, where we found reduced binding with IκBα, which might be a consequence of IκBα degradation, that the further assessment with PPL pretreatment followed by LPS stimulation led to an increased association of both p65 and HDAC3 proteins with IκBα. These findings are also consistent with the docking studies, where PPL was found to well lodge at the interface of the p65 NF-κB/IκBα complex (Fig. 8).

There is a high demand for antipsoriatic therapies with long-term safety, good tolerability, ease of use, and high efficacy with limited adverse effects. Both in vitro and preclinical evidences demonstrated for the first time that PPL exhibits potent anti-proliferative and anti-inflammatory activities with negligible adverse effects, making it a potentially translational promise to address the clinically unmet needs of psoriasis. Further investigations are needed to explore its clinical outcomes.

## Materials and methods

### Chemicals

Piperlongumine (PPL) was purchased from AK Scientific California, USA. EGF recombinant human protein was purchased from Thermo Fisher Scientific, USA. MTT, AO, EB, DAPI, DCFDA, JC-1, LPS from *Escherichia coli*, protease inhibitor cocktail, Poly-L-lysine solution, FragEL™ DNA Fragmentation Detection Kit, and Fluorescent-TdT Enzyme Assay Kit were procured from Sigma-Aldrich, USA. Protein A/G Magnetic Beads were purchased from Invitrogen, USA. IMQ cream, 5% w/w was purchased from Glenmark Pharmaceuticals India. Nitrocellulose membrane, ECL reagent, and protein dual marker were procured from Bio-Rad, USA. MILLIPLEX MAP Kit was obtained from Millipore, Germany. Enzyme-linked immunosorbent assay kits for IL-1β, IL-6, IL-17A, IL-22, TNF-α, and TGF-β were procured from Thermo Fisher Scientific, USA. All the chemicals used in the present study were pure and of analytical grade.

### Antibodies

p-Akt (Ser473) (#4060), p-Akt (Thr308) (#13038), Akt (#4691), Cyclin D1 (#2978), p-p38 (Thr180/Tyr182) (#4511), p-ERK1/2 (Thr202/Tyr204) (#4370), ERK1/2 (#9102), p38 (#8690), Bcl-2 (#3498), K17 (#4543), p-IKKα/β (Ser176/180) (#2697), IKKβ (#8943), p-IκBα (Ser32) (#2859), p-p65 (Ser536) (#3033), p65 (#8242), p-p70S6K (#9234), p70S6K (#9202), HDAC1 (#5356), HDAC2 (#5113), HDAC3 (#3949), HDAC4 (#7628), HDAC6(#7558), H3 (#4499), p-SAPK/JNK (Thr183/Tyr185) (#4668), and SAPK/JNK (#9252) antibodies were purchased from Cell Signaling Technologies, USA. mTOR (# sc-1549), ICAM-1 (#sc-8439), β-Actin (#sc-47778), COX-2 (#sc-1745), PCNA (#sc-7907), Ki-67 (#sc-15402), p-STAT3 (#sc-8059), STAT3 (#sc-8019), anti-Goat (#Sc-2354), anti-mouse (#Sc-2061), and anti-mouse (#Sc-2030) IgG-horseradish peroxidase (HRP) antibodies were procured from Santa Cruz Biotechnology, USA.

### Cell culture

The spontaneously immortalized human epidermal keratinocytes (HaCaT) cell line was a kind gift sample from Dr. Munia Ganguli, Institute of Genomics and Integrative Biology, New Delhi, India. Mouse macrophages (RAW 264.7) were procured from National Centre for Cell Science, Pune, India. Both the cell lines were cultured in appropriate RPMI-1640 or Dulbecco’s modified Eagle’s medium, respectively, supplemented with 1% antibiotic and anti-mycotic solution (Sigma-Aldrich, USA) and 10% fetal bovine serum (Invitrogen, Gibco, USA). Cells were trypsinized (0.25% trypsin-EDTA solution; Invitrogen, USA) and sub-cultured when attained 70–80% confluence. Ten mM stock solution of PPL was prepared in dimethyl sulfoxide and further dilutions were made in the respective media. Both the cell lines were authenticated by STR profiling and tested for mycoplasma contamination.

### Cell viability

For analyzing the effect of PPL on cell proliferation of HaCaT and RAW 264.7 cells, MTT assay was performed as described earlier with slight modifications^65^.

### Fluorescence microscopic examination of apoptosis

PPL-induced morphological and apoptotic changes were observed by AO/EB and DAPI staining. Briefly, HaCaT cells were seeded in 12-well plate, and the next day, cells were treated with PPL at the concentrations of 2.5, 5, and 10 µM. After 2 h, cells were stimulated with EGF (50 ng/ml) and further incubated for 48 h, then stained with AO/EB (10 µg/ml each) mixture for 10 s and washed twice with phosphate-buffered saline (PBS). For DAPI staining, cells were fixed with 4% paraformaldehyde and permeabilized with 0.1% Triton X-100, then cells were stained with DAPI (1 µM) in PBS, and the images were captured at ×200 magnification by fluorescent microscope (Nikon Eclipse TiS, Japan)^66^.

### Determination of cell cycle analysis

HaCaT cells were cultured and treated with PPL for 2 h and incubated with EGF for 48 h. Later, the cells were collected by trypsinization and fixed in 70% ethanol and stored at −20 °C. Later, cells were washed with PBS and incubated with PI staining buffer (a mixture of PI, triton-X-100, RNAse enzyme, and double distilled water) for 15 min and then subjected to flow cytometric analysis (BD C6 Accuri flow cytometry, USA)^67^.

### JC-1 staining

∆Ψm loss leads to the ROS-dependent activation of apoptosis, where JC-1 reversibly forms “J aggregates” with red fluorescence at high membrane potential, while during ∆Ψm loss it forms green fluorescence due to “J monomers.” To perform the assay, HaCaT cells were seeded in 12-well plates and cells were treated PPL and stimulated with EGF, respectively. After 48 h of incubation, cells were stained with JC-1 dye at 1 µM for 30 min and subjected to flow cytometry^68^.

### Alexa Fluor 488 Annexin V/Dead Cell apoptosis assay

Alexa Fluor® 488 Annexin V/Dead Cell Apoptosis kit (Thermo Fisher Scientific, USA) was used to evaluate the apoptosis induction, quantitatively. Here cells were seeded in 12-well plates and treated with PPL for 2 h and incubated with EGF for 24 h. Then cells were harvested and suspended by 500 μl binding buffer and incubated with 5 μl Alexa Fluor 488 and 1 μl PI for 15 min at room temperature (RT) and the quantitative analysis was performed by flow cytometry^69^.

### TUNEL assay

Apoptotic cells were visualized by using the TUNEL assay kit. Cells were cultured on confocal chamber slides and then fixed with 4% paraformaldehyde in PBS. Then cells were treated with proteinase K (20 mg/ml in PBS) for 15 min at RT. The samples were washed with PBS again and processed using a FragEL™ DNA Fragmentation Detection Kit, Fluorescent-TdT Enzyme (Calbiochem) according to the manufacturer’s instructions. The samples were then counter-stained with DAPI. TUNEL positivity in cells was captured by a confocal microscope at ×400 magnification.

### Multiplex analysis

RAW 264.7 cells were pretreated with PPL for 2 h and later cells were stimulated with LPS (1 µg/ml) for 24 h. Later, protein was isolated with RIPA lysis buffer, and inflammatory cytokines and chemokines such as IFN-γ, IL-1β, IL-2, IL-3, IL-6, IL-10, IL-17A, TNF-α, MCP-1, MIP, MIP-1α and -1β levels were measured by Luminex assay based on xMAP technology (MAGPIX, Millipore, Germany). This assay was conducted with a customized highly sensitive MILLIPLEX MAP kit (Millipore, Germany) according to the manufacturer’s protocol.

### Animal study and ethical statement

The animal study was performed in 6–8 week-old male BALB/c mice (weight 25–30 g). Mice were procured from Palamuru eBiosciences Pvt. Ltd, Mahabubnagar, India. Animals were housed and acclimatized at least 1 week. All procedures of the study were approved by the Institutional Animal Ethics Committee (Approval No: NIP/7/2017/RT/237), NIPER-Hyderabad, India. All the experiments were conducted in accordance with the Committee for the Purpose of Control and Supervision of Experiments on Animals (CPCSEA) guidelines, Government of India.

### IMQ-induced psoriasis-like skin inflammation model and protective effect of PPL

Psoriasis like skin inflammation was developed in BALB/c mice at a daily dose of 3.125 mg of the active compound in 62.5 mg lotion/day per 5 cm^2^ area on to the shaven dorsal region of the skin and the left ear for 6 consecutive days. The mice were randomized into six different groups with *n* = 5 per group. Normal control (NC) group, IMQ control, PL, and PH group animals were applied topical PPL treatment once daily (PL-10 and PH-30 mg/kg); PSC group received PPL 1 mg/kg subcutaneously as daily injection. Here the TAC group served as standard, where 50 mg gel that contains 20 mg/kg TAC was applied topically. Carbopol gel was prepared by neutralizing the gel to pH 6 by drop-wise addition of triethanolamine and the gel was allowed to swell completely followed by the addition of PPL to accurately weighed amount of different percentages of gel. The gels were evaluated for rheological behavior and spreadability using modular compact rheometer (Anton-Paar, USA). For PPL topical application, carbopol 0.75% was chosen as the matrix for adequate viscosity and ease in application. PPL 10 and 30 mg/kg were mixed in the gel and applied from day 3 and continued till day 6, post 4 h IMQ application. The psoriasis area severity index (PASI) for erythema, scaling, and thickening was graded independently from 0 to 4 as follows: 0, none; 1, slight; 2, moderate; 3, marked; 4, very marked. Every alternate day, skin fold and ear fold thickness were measured by Vernier calipers (M & W Precision Tools, India).

### Spleen to body weight index

Animal body weights from all groups were recorded till the termination of the study and the spleen from each mouse was isolated and a photograph was taken before weighing. The spleen weights were normalized with body weights to obtain organ index (Spleen weight/Body weight) and results are expressed in mg/g.

### Enzyme-linked immune sorbent assay

Skin tissues were homogenized in ice-cold lysis buffer and protein was isolated as per the protocol described previously to determine the various inflammatory cytokines, such as IL-1β, IL-6, IL-17A, IL-22, TGF-β, and TNF-α (Thermo Fisher Scientific, USA). The assay was performed as per the manufacturer’s guidelines. The cytokine levels were normalized by the Bradford protein assay. Results are expressed as pg/mg protein^70^.

### HDAC fluorometric assay

HDAC inhibitory activity of PPL was measured by the Histone Deacetylase Assay Kit, Fluorometric (Sigma-Aldrich, USA), which was based on a two-step enzymatic reaction. Here various concentrations of PPL (0.05, 0.1, 1, and 2.5 µM) were incubated with the HeLa cell lysate and substrate having acetylated lysine residues and bound fluorescent groups, which were provided along with kit. After 30 min incubation, developer solution was added to the above components and further incubated for 10 min. Later, the fluorescence was measured with the multimode plate reader (Spectramax M4, Molecular Devices, USA). The HDAC inhibitor TSA was used as a standard to compare the HDAC inhibitory activity of PPL from the standard curve plotted from the non-acetylated substrate (standard), which was provided in the kit.

### Histopathological studies

Mouse skin tissues were preserved in 10% formalin and stored at RT until tissue sectioning. Then tissues were dehydrated by incubating with gradient alcohols and xylene. Five-micron skin sections were sliced by microtome (Leica, Germany). Then skin tissue sections were collected on poly-L-lysine (Sigma-Aldrich, USA) coated slides. H & E (Sigma-Aldrich, USA) staining was used to differentiate the nucleus and cytoplasm. This staining was performed to evaluate the epidermal hyperplasia and skin inflammation. After the staining, the images were captured by a bright-field microscope at ×200 or ×400 magnifications. In support of H & E staining, the keratinocyte hyperproliferation was determined by DAPI nuclear staining, and the images were captured by a confocal microscopy at ×400 magnification. The epidermal thickness was measured based on the picture of the H & E images by the NIKON NIS Elements software.

### IF analysis

HaCaT and RAW 264.7 cells were seeded in cell imaging chamber slides (Eppendorf, Germany) and treated with PPL in the presence of EGF or LPS, respectively. Later, cells were fixed with paraformaldehyde and permeabilized with Triton X-100. Then cells were blocked for 1 h and probed with relevant STAT3, K17, p65, and HDAC3 antibodies at 1:200 dilutions, whereas for skin tissue *s*amples, 5 µm size sections were de-waxed for 20 min at 65 °C and treated with xylene and rehydrated in a graded series of ethanol. The skin sections were subjected to proteinase-K for antigen retrieval. Later, sections were blocked with 3% bovine serum albumin (BSA) for 1 h at RT, immunostained with primary antibodies STAT3, K17, p65, and HDAC3 (1:100 dilutions) overnight at 4 °C, and probed with fluorescence-conjugated fluorescein isothiocyanate or rhodamine secondary antibodies followed by washing and mounting with Fluoroshield™ with DAPI histology mounting medium (Sigma-Aldrich, USA). Images were captured using Leica TCS SP8 Laser Scanning Spectral Confocal Microscope.

### Western blot analysis

The whole-cell lysate and nuclear and cytoplasmic extracts from cells and skin tissues were extracted with RIPA buffer with protease and phosphatase inhibitors. The protein levels were quantified and normalized by the bicinchonic acid kit (Sigma-Aldrich, USA). Cells and skin tissues were homogenized in cell lysis buffer. The protein samples were subjected to sodium dodecyl sulfate-polyacrylamide gel electrophoresis (SDS-PAGE). The proteins were transferred from gel to nitrocellulose membrane by wet transfer apparatus (Bio-Rad, USA). After blocking (3% BSA), the membrane was probed with primary and secondary antibodies. The protein expression was determined by ECL and blots were captured by Chemdoc system (Vilber Fx, France). All the protein levels were normalized by respective totals, β-Actin, or H3 and quantified by the ImageJ, NIH, USA software as per the detailed procedure given in our previous work^11^.

### Cytoplasmic Co-IP

Cytoplasmic extracts were prepared and the primary antibody IκBα or control rabbit preimmune IgG were coupled with Dynabeads Protein G beads (Invitrogen, Carlsbad, CA) and incubated overnight at 4 °C. The antibody–antigen immunocomplex was collected through magnetic separation and washed with PBS for three times. Immunocomplexes were released by boiling in 2× SDS sample buffer. Proteins were resolved on 10% SDS gel and blocked with 3% BSA and the membrane was incubated with the primary antibodies p65 and HDAC3 overnight, followed by incubation with an HRP-conjugated secondary antibody for 1 h. The signal was visualized by Chemdoc system^71^.

### Skin compliance and safety evaluation of PPL alone on topical and subcutaneous route of administration

Male BALB/c mice were randomized and divided into normal control (NC only gel), PPL topically (PL = 30 mg/kg in gel), and PPL subcutaneous in saline (PSC = 1 mg/kg) with *n* = 3 per group. Mice from all the groups were dehaired on the dorsal region with a topical depilatory, after 2 days PPL 30 mg/kg (equivalent to the highest dose tested on IMQ-applied skin) was incorporated in the carbopol gel and applied topically every day on to the shaved back of the skin, while PPL 1 mg/kg was injected daily in subcutaneous route for 7 and 21 days. Mice were monitored daily for the signs and symptoms such as erythema, papules, or any type of phenotypic changes in the skin including the body weight reduction. Animals were sacrificed on days 7 and 21 and analyzed further for histopathological studies and immunoblotting.

### Molecular docking

Molecular modeling was performed using bioinformatic tools to find molecular interactions of PPL on p65-IκBα-HDAC3 complex. Detailed procedure is described in Supplementary Data.

### Statistical analysis

The results are expressed as mean ± standard deviation (SD) and *n* refers to the number of sample replicates. One-way analysis of variance was applied along with Bonferroni post hoc test for statistical analysis. Prism software (version 6.01; GraphPad, USA) was used to analyze the data and *P* < 0.05 was considered to be statistically significant.

## Supplementary information

Supplementary data

Supplementary data

Supplementary data

Supplementary data

Supplementary data

Supplementary data

Supplementary data

Supplementary data

Supplementary data

Supplementary data

Supplementary data

Supplementary data

Supplementary data

Supplementary data

Supplementary data

## References

[1] Albanesi. C, Madonna S, Gisondi P, Girolomoni G (2018). The interplay between keratinocytes and immune cells in the pathogenesis of psoriasis. Front. Immunol..

[2] Lowes MA, Bowcock AM, Krueger JG (2007). Pathogenesis and therapy of psoriasis. Nature.

[3] Wang S, Zhang Z, Peng H, Zeng K (2019). Recent advances on the roles of epidermal growth factor receptor in psoriasis. Am. J. Transl. Res..

[4] Chen W, Wu L, Zhu W, Chen X (2018). The polymorphisms of growth factor genes (VEGFA & EGF) were associated with response to acitretin in psoriasis. Per. Med..

[5] Li HH (2005). Interleukin-19 upregulates keratinocyte growth factor and is associated with psoriasis. Br. J. Dermatol..

[6] Eding CB, Enerback C (2017). Involved and uninvolved psoriatic keratinocytes display a resistance to apoptosis that may contribute to epidermal thickness. Acta Derm. Venereol..

[7] Madonna S, Scarponi C, Pallotta S, Cavani A, Albanesi C (2012). Anti-apoptotic effects of suppressor of cytokine signaling 3 and 1 in psoriasis. Cell Death Dis..

[8] Jin L, Wang G (2014). Keratin 17: a critical player in the pathogenesis of psoriasis. Med. Res. Rev..

[9] Mavropoulos, A., Rigopoulou, E. I., Liaskos, C., Bogdanos, D. P. & Sakkas, L. I. The role of p38 MAPK in the aetiopathogenesis of psoriasis and psoriatic arthritis. *Clin. Dev. Immunol.* **2013**, 569751 (2013). 10.1155/2013/569751 PMC3787653 24151518

[10] Leoni F (2002). The antitumor histone deacetylase inhibitor suberoylanilide hydroxamic acid exhibits antiinflammatory properties via suppression of cytokines. Proc. Natl Acad. Sci. USA.

[11] Pooladanda V (2019). Nimbolide protects against endotoxin-induced acute respiratory distress syndrome by inhibiting TNF-α mediated NF-κB p65and HDAC-3 nuclear translocation. Cell Death Dis..

[12] Wu WP (2017). The attenuation of renal fibrosis by histone deacetylase inhibitors is associated with the plasticity of FOXP3+IL-17+ T cells. BMC Nephrol..

[13] Glauben R, Sonnenberg E, Wetzel M, Mascagni P, Siegmund B (2014). Histone deacetylase inhibitors modulate interleukin 6-dependent CD4+ T cell polarization in vitro and in vivo. J. Biol. Chem..

[14] Kiernan R (2003). Post-activation turn-off of NF-κB-dependent transcription is regulated by acetylation of p65. J. Biol. Chem..

[15] Zhu H, Shan L, Schiller PW, Mai A, Peng T (2010). Histone deacetylase-3 activation promotes tumor necrosis factor-α (TNF-α) expression in cardiomyocytes during lipopolysaccharide stimulation. J. Biol. Chem..

[16] Durham BS, Grigg R, Wood IC (2017). Inhibition of histone deacetylase 1 or 2 reduces induced cytokine expression in microglia through a protein synthesis independent mechanism. J. Neurochem..

[17] Gottlieb AB (2005). Psoriasis: emerging therapeutic strategies. Nat. Rev. Drug. Discov..

[18] van der Fits L (2009). Imiquimod-induced psoriasis-like skin inflammation in mice is mediated via the IL-23/IL-17 axis. J. Immunol..

[19] Horváth S (2019). Methodological refinement of Aldara-induced psoriasiform dermatitis model in mice. Sci. Rep..

[20] Prasad S, Tyagi AK (2016). Historical spice as a future drug: therapeutic potential of piperlongumine. Curr. Pharm. Des..

[21] Bezerra DP (2013). Overview of the therapeutic potential of piplartine (piperlongumine). Eur. J. Pharm. Sci..

[22] Na Takuathung M (2018). Antipsoriatic effects of wannachawee recipe on imiquimod-induced psoriasis-like dermatitis in BALB/c mice. Evid. Based Complement. Alternat. Med..

[23] Chapman, J. & Azevedo, A. M. *Splenomegaly* (StatPearls Publishing, 2019). 28613657

[24] Lin ZM (2018). Topical administration of reversible SAHH inhibitor ameliorates imiquimod-induced psoriasis-like skin lesions in mice via suppression of TNF-α/IFN-γ-induced inflammatory response in keratinocytes and T cell-derived IL-17. Pharmacol. Res..

[25] Yang J (2009). Expression of antiapoptotic protein c-FLIP is upregulated in psoriasis epidermis. Eur. J. Dermatol..

[26] Flisiak I, Szterling-Jaworowska M, Baran A, Rogalska-Taranta M (2014). Effect of psoriasis activity on epidermal growth factor (EGF) and the concentration of soluble EGF receptor in serum and plaque scales. Clin. Exp. Dermatol..

[27] Nanney LB, Stoscheck CM, Magid M, King LE (1986). Altered [125I] epidermal growth factor binding and receptor distribution in psoriasis. J. Invest. Dermatol..

[28] Elango T (2017). Methotrexate treatment provokes apoptosis of proliferating keratinocyte in psoriasis patients. Clin. Exp. Med..

[29] Chamcheu JC (2017). Dual inhibition of PI3K/Akt and mTOR by the dietary antioxidant, delphinidin, ameliorates psoriatic features in vitro and in an imiquimod-induced psoriasis-like disease in mice. Antioxid. Redox Signal..

[30] Shi X (2011). IL-17A upregulates keratin 17 expression in keratinocytes through STAT1- and STAT3-dependent mechanisms. J. Invest. Dermatol..

[31] Clark RA, Kupper TS (2006). Misbehaving macrophages in the pathogenesis of psoriasis. J. Clin. Invest..

[32] Wang H (2006). Activated macrophages are essential in a murine model for T cell–mediated chronic psoriasiform skin inflammation. J. Clin. Invest..

[33] Nakai K (2017). IL-17A induces heterogeneous macrophages, and it does not alter the effects of lipopolysaccharides on macrophage activation in the skin of mice. Sci. Rep..

[34] Dorrington, M. G. & Fraser, I. D. C. NF-κB p65 signaling in macrophages: dynamics, crosstalk, and signal integration. *Front. Immunol*. **10**, 705 (2019). 10.3389/fimmu.2019.00705 PMC6465568 31024544

[35] Kesanakurti D, Chetty C, Maddirela DR, Gujrati M, Rao JS (2013). Essential role of cooperative NF-κB and Stat3 recruitment to ICAM-1 intronic consensus elements in the regulation of radiation-induced invasion and migration in glioma. Oncogene.

[36] Grabiec AM (2010). Histone deacetylase inhibitors suppress inflammatory activation of rheumatoid arthritis patient synovial macrophages and tissue. J. Immunol..

[37] Camelo S (2005). Transcriptional therapy with the histone deacetylase inhibitor trichostatin A ameliorates experimental autoimmune encephalomyelitis. J. Neuroimmunol..

[38] Li N (2008). HDAC inhibitor reduces cytokine storm and facilitates induction of chimerism that reverses lupus in anti-CD3 conditioning regimen. Proc. Natl Acad. Sci. USA.

[39] Place RF, Noonan EJ, Giardina C (2005). HDAC inhibition prevents NF-kappa B activation by suppressing proteasome activity: down-regulation of proteasome subunit expression stabilizes I kappa B alpha. Biochem. Pharmacol..

[40] Gao Z, He Q, Peng B, Chiao P, Ye J (2006). Regulation of nuclear translocation of HDAC3 by IkBalpha is required for TNF-inhibition of PPARgamma function. J. Biol. Chem..

[41] Manczinger, M. & Kemény, L. Novel factors in the pathogenesis of psoriasis and potential drug candidates are found with systems biology approach. *PLoS ONE* **8**, e80751 (2013). 10.1371/journal.pone.0080751 PMC3841158 24303025

[42] Viatour P (2003). Cytoplasmic IκBα increases NF-κB-independent transcription through binding to histone deacetylase (HDAC) 1 and HDAC3. J. Biol. Chem..

[43] Wraight CJ (2000). Reversal of epidermal hyperproliferation in psoriasis by insulin-like growth factor I receptor antisense oligonucleotides. Nat. Biotechnol..

[44] Ruckert R (2000). Inhibition of keratinocyte apoptosis by IL-15: a new parameter in the pathogenesis of psoriasis?. J. Immunol..

[45] Ogawa E, Sato Y, Minagawa A, Okuyama R (2018). Pathogenesis of psoriasis and development of treatment. J. Dermatol..

[46] Moorchung N, Vasudevan B, Kumar SD, Muralidhar A (2015). Expression of apoptosis regulating proteins p53 and bcl-2 in psoriasis. Indian J. Pathol. Microbiol..

[47] Wrone-Smith T (1997). Keratinocytes derived from psoriatic plaques are resistant to apoptosis compared with normal skin. Am. J. Pathol..

[48] Pietrzak A, Miturski R, Krasowska D, Postawski K, Lecewicz-Torun B (1999). Concentration of an epidermal growth factor in blood serum of males during topical treatment of psoriasis. J. Eur. Acad. Dermatol. Venereol..

[49] Szterling-Jaworowska M, Baran A, Myśliwiec H, Flisiak I (2018). Effect of psoriasis activity and topical treatment on plasma epidermal growth factor (EGF) and its soluble receptor (sEGFR). J. Dermatol. Treat..

[50] Szterling-Jaworowska M, Flisiak I, Baran A, Chodynicka B (2009). The role of epidermal growth factor in psoriasis. Przegl. Dermatol..

[51] Bruner CR, Feldman SR, Ventrapragada M, Fleischer AB (2003). A systematic review of adverse effects associated with topical treatments for psoriasis. Dermatol. Online J..

[52] Weidmann A, Foulkes AC, Kirkham N, Reynolds NJ (2014). Methotrexate toxicity during treatment of chronic plaque psoriasis: a case report and review of the literature. Dermatol. Ther..

[53] Yang L, Fan X, Cui T, Dang E, Wang G (2017). Nrf2 promotes keratinocyte proliferation in psoriasis through up-regulation of keratin 6, keratin 16, and keratin 17. J. Invest. Dermatol..

[54] Kim Hyung-Ran, Lee Anbok, Choi Eun-Jeong, Hong Min-Pyo, Kie Jeong-Hae, Lim Woosung, Lee Hyeon Kook, Moon Byung-In, Seoh Ju-Young (2014). Reactive Oxygen Species Prevent Imiquimod-Induced Psoriatic Dermatitis through Enhancing Regulatory T Cell Function. PLoS ONE.

[55] Bai F (2017). Serum levels of adipokines and cytokines in psoriasis patients: a systematic review and meta-analysis. Oncotarget.

[56] Ghoreschi K, Weigert C, Röcken M (2007). Immunopathogenesis and role of T cells in psoriasis. Clin. Dermatol..

[57] Xiao Y (2016). Piperlongumine suppresses dendritic cell maturation by reducing production of reactive oxygen species and has therapeutic potential for rheumatoid arthritis. J. Immunol..

[58] Tung, K. Y. et al. in *Epigenetics and Dermatology* (eds Lu, Q., Chang, C. C. & Richardson, B. C.) Ch. 11 (Academic Press, 2015).

[59] Pollock RA, Abji F, Gladman DD (2017). Epigenetics of psoriatic disease: a systematic review and critical appraisal. J. Autoimmun..

[60] Tovar‐Castillo LE (2007). Under-expression of VHL and over-expression of HDAC-1, HIF-1alpha, LL-37, and IAP-2 in affected skin biopsies of patients with psoriasis. Int. J. Dermatol..

[61] Leus NG (2016). HDAC 3-selective inhibitor RGFP966 demonstrates anti-inflammatory properties in RAW 264.7 macrophages and mouse precision-cut lung slices by attenuating NF-κB p65 transcriptional activity. Biochem. Pharmacol..

[62] Chen X (2012). Requirement for the histone deacetylase Hdac3 for the inflammatory gene expression program in macrophages. Proc. Natl Acad. Sci. USA.

[63] Ge Z (2013). Vorinostat, a histone deacetylase inhibitor, suppresses dendritic cell function and ameliorates experimental autoimmune encephalomyelitis. Exp. Neurol..

[64] Komives, E. A. *Consequences of Fuzziness in the NF-κB/IκBκ Interaction* (Springer, New York, NY, 2012).

[65] Sunkari S, Thatikonda S, Pooladanda V, Challa VS, Godugu C (2019). Protective effects of ambroxol in psoriasis like skin inflammation: exploration of possible mechanisms. Int. Immunopharmacol..

[66] Tokala R (2018). Synthesis of 1,2,4-triazole-linked urea/thiourea conjugates as cytotoxic and apoptosis inducing agents. Bioorg. Med. Chem. Lett..

[67] Nekkanti S (2017). Synthesis of 1,2,3-triazolo-fused-tetrahydro-β-carboline derivatives via 1,3-dipolar cycloaddition reaction: cytotoxicity evaluation and DNA-binding studies. Chem. Sel..

[68] Kumar NP (2018). Synthesis of enamino-2-oxindoles via conjugate addition between α-azido ketones and 3-alkenyl oxindoles: cytotoxicity evaluation and apoptosis inducing studies. Bioorg. Med. Chem. Lett..

[69] Pooladanda V, Bandi S, Mondi SR, Gottumukkala KM, Godugu C (2018). Nimbolide epigenetically regulates autophagy and apoptosis in breast cancer. Toxicol. In Vitro.

[70] Bale S, Venkatesh P, Sunkoju M, Godugu C (2018). An adaptogen: withaferin A ameliorates in vitro and in vivo pulmonary fibrosis by modulating the interplay of fibrotic, matricelluar proteins, and cytokines. Front. Pharmacol..

[71] Antrobus R, Borner GHH (2011). Improved elution conditions for native co-immunoprecipitation. PLoS ONE.

