# Supplementary material for: Piperlongumine regulates epigenetic modulation and alleviates psoriasis-like skin inflammation via inhibition of hyperproliferation and inflammation
Source: Cell Death Dis. 2020 Jan 10;11(1):21. doi: 10.1038/s41419-019-2212-y (PMC6954241; doi:10.1038/s41419-019-2212-y)
Supplement: Supplementary file 1 — Supplementary data [file 41419_2019_2212_MOESM1_ESM.docx]

**Piperlongumine regulates epigenetic modulation and alleviates psoriasis like skin inflammation** **via inhibition of hyperproliferation and inflammation** Sowjanya Thatikonda^1^, Venkatesh Pooladanda^1^, Dilep Kumar Sigalapalli^2^, Chandraiah Godugu^1*^

^1^Department of Regulatory Toxicology, National Institute of Pharmaceutical Education and Research (NIPER), Balanagar, Hyderabad, Telangana-500037, India.

^2^Department of Medicinal Chemistry, National Institute of Pharmaceutical Education and Research (NIPER), Balanagar, Hyderabad, Telangana-500037, India.

**Running title:** Piperlongumine ameliorates psoriasis like skin inflammation

^*^Correspondence:

Dr. Chandraiah Godugu, Assistant Professor,

Department of Regulatory Toxicology,

National Institute of Pharmaceutical Education and Research (NIPER),

Balanagar, Hyderabad,

Telangana, India-500037

Telephone: 040-23073741 Fax: 040-23073751

E-mail: [chandragodugu@gmail.com](mailto:chandragodugu@gmail.com),

[chandra.niperhyd@gov.in](mailto:chandra.niperhyd@gov.in)

**1. Supplementary methods**

**1.1. Molecular docking studies**

The three-dimensional PDB coordinates for p65/IκBα (PDB ID: 1IKN) protein complex and to facilitate the HDAC3/IκBα protein complex, the 3D crystal structures of proteins: HDAC3 (PDB ID: 4A69) and IκBα (PDB ID: 1IKN) were retrieved from the RCSB Protein Data Bank^1,2^. First, the HDAC3/IκBα protein complex was mimicked by docking HDAC3 and IκBα using protein-protein docking module of Schrödinger software^3^. The protein complexes p65/IκBα and HDAC3/IκBα were prepared using Protein Preparation Wizard of Maestro. Hydrogen atoms were added, appropriate protonation states were assigned to all the residues using Epik. Missing amino acid residues were modelled using Prime^4,5^. The prepared protein complexes were further subjected to SiteMap computer program to identify their potential binding sites. The ligand molecule was built on Maestro Molecule Builder of Schrödinger. The built ligand was optimized using OPLS-2005 force field in LigPrep module of Schrödinger software. All possible protomers and ionization states were enumerated for ligand using Ionizer at a pH of 7.4. Tautomeric states were generated for chemical groups with possible prototropic tautomerism.

Molecular docking studies were performed by using a GLIDE docking module of Schrödinger suite. The prepared ligand was docked into the generated receptor grids using Glide SP docking precision. Each complex was analyzed for interactions and the 3D poses were taken. 3D poses demonstrates the molecular recognition interactions. All the 3D figures were obtained using Schrödinger suite. The MM/GBSA (Molecular mechanics/generalized born surface area) analysis was used to calculate ligand-binding energies based on docking complex, using the MM/GBSA technology available in Prime module of Schrodinger software. The protein ligand complexes obtained from molecular docking were subjected to MM/GBSA calculations.

**2. Supplementary results**

**2.1. Identification of binding sites on protein complexes using SiteMap analysis**

SiteMap combines a novel and highly effective algorithm for rapid binding site identification for various proteins^3–5^. **Tables S1** and S**2** depicts the SiteMap analysis results for top most potential binding sites of p65/IκBα and HDAC3/IκBα protein complexes. From the SiteMap analysis we have observed, Site-IV of p65/IκBα complex and Site-I of HDAC3/IκBα has the top most Site score (ability to bind a ligand) and D score (druggability). Predicted binding sites with Site Score and D Score greater than 1.1 are classified as highly druggable binding sites. Hydrophilic (Hydrophilic character of the binding site) and Hydrophobic (Hydrophobic character of the binding site) scores of Site-IV and Site-I is convincingly excellent. Results illustrate the identified potential binding sites on p65/IκBα **(Figure 7b, c)** and HDAC3/IκBα **(Figure 7e, f)** protein complexes respectively. Further, molecular docking and Prime^3^ MM/GBSA binding energy calculations were performed for PPL on Site-IV of p65/IκBα and Site-I of HDAC3/IκBα protein complex.

**2.2. Molecular docking and Prime MM/GBSA binding energy calculations**

Protein-protein interactions play a key role in a variety of diseases including psoriasis. Druggable potential binding sites that present at protein-protein interfaces could be helpful to develop small molecules as drug candidates. Interestingly, the identified potential binding sites, Site-IV and Site-I lies at the interfaces of p65/IκBα and HDAC3/IκBα protein complexes, respectively. In this aspect, molecular docking and Prime MM/GBSA binding energy calculations were performed to explore the binding mode, type of interactions and binding energy between ligand-protein complexes using Glide docking module^3,6,7,8^. **Table S3** demonstrates the result of the molecular docking along with H-bonding as well as hydrophobic and arene-arene interactions of PPL with p65/IκBα and HDAC3/IκBα protein complexes.

From the molecular docking analysis, it was observed that the top ranked conformation of PPL was well lodged at the interface of p65/IκBα complex. PPL has shown three hydrogen bond interactions with the binding site residues Arg143, Asn145 and Arg253. **Figure 7b, c** revealed various hydrogen bonding, **π-π** stacking interactions that appear to play a key role in the binding mode. The 4-methoxy group of 3,4,5-trimethoxy phenyl moiety acts as hydrogen bond acceptor and involved in H-bond interaction with side chain NH_2_ of Arg253 (d = 3.00 Å). The carbonyl functional group of acryloyl moiety has formed a two point hydrogen bond contact with the binding site amino acid residues Arg143, Asn145 with a distance of 3.26 Å and 2.59 Å, respectively. Similarly the oxygen atom of dihydro-pyridinone had shown hydrogen bonding interaction with side chain NH_2_ of Arg143 (d = 1.77 Å). The trimethoxyphenyl moiety of PPL also had two **π-π** stacking interactions with the binding site residues His184 and Arg253. Additionally, several hydrophobic interactions were observed between PPL and the binding site residues, e.g., Leu189, Ile192, Cys215, Leu223 and Leu227 are stabilizes the binding of the PPL in the interface of p65/IκBα complex.

Furthermore, molecular docking simulation studies were also performed to enumerate the binding mode of PPL into the binding site (Site-I) of HDAC3/IκBα protein complex^3^ illustrate the docking pose of PPL at the interface HDAC3/IκBα protein complex **(Figure 7e, f)**. PPL formed three hydrogen bond contacts with the binding site residues Cys167, Val300 and Arg301. The 4-methoxy group of 3,4,5-trimethoxy phenyl moiety acts as hydrogen bond acceptor and involved in H-bond interaction with back bone NH_2_ of Cys167 with a distance of 2.21 Å. The carbonyl functional group of acryloyl moiety has formed a hydrogen bond interaction with Val300 (d = 2.14Å). Next, the oxygen atom of dihydro-pyridinone has formed a strong back bone hydrogen bond interaction with the binding site amino acid residue Arg301 (d = 2.04 and 2.58 Å). Several hydrophobic interactions were also observed between PPL and the binding site residues Leu130, Leu131, Ala133, Cys167, Cys263, Tyr298, Val300 and Tyr331. All these interactions certainly stabilize the binding of PPL in the interface binding site of the HDAC3/IκBα protein complex_._

**2.3. Safety evaluation of PPL alone on topical and subcutaneous routes of administration**

Skin compliance and dermal safety evaluation study was performed on healthy male BALB/c mice to determine the compatibility of PPL on topical and subcutaneous routes of administration and to observe any type of phenotypic signs which include the erythema, papules formation or acute inflammatory responses. These changes were monitored by daily topical administration of PPL at 30 mg/kg dose (PH) and daily subcutaneous administration of PPL at 1 mg/kg dose (PSC) for 7 and 21 days. The body weight changes were monitored every alternate day throughout the study. No significant signs or toxicity was observed in either of the route for 7 days and the animals appeared normal and intact **(Figure S2a)**, then the animals were scarified and the skin tissues were collected and stored at -80 ℃, whereas for histopathological analysis skin tissues were stored at 10% formalin. On the other side, daily treatment was carried out up to 21 days. In topical route upon continuous administration of PPL, dry and dehydrated skin was observed in topical route treatment from the 12^th^ day of administration, while no marked changes were observed with SC treatment. Other signs like erythema, or inflammatory responses were not observed up to 21 days in both the routes, on the 22^nd^ day of sacrifice mild dryness was observed with no change in body weights and other notable symptoms **(Figure S3a)**. Animals were sacrificed and observed for the anatomical changes in the skin. The H & E staining from the 7^th^ day animals group exhibited mild reduction in the epidermis in PSC group which is not significant **(Figure S2b, c)**, while immunoblotting analysis showed moderate but not significant increase in STAT3 phosphorylation in PSC group, no significant changes were observed in p65 phosphorylation **(Figure S2d-f)**. On the other hand, the results from the 21 days treated animals H & E staining; a mild decrease in the epidermal thickness measurement was observed **(Figure S3b).** However, these changes were insignificant in both groups. Similar to 7 days treatment, immunoblotting showed minimal increase in STAT3 phosphorylation in PSC group, while a slight decrease in p65 phosphorylation in PH treatment and these changes are not significant in either of the groups **(Figure S3d-f)**.

**2.4. PPL alone treatment at longer duration on keratinocytes and macrophages**

MTT assay was performed in RAW 264.7 and HaCaT cells to assess the cell viability of PPL alone treatment without stimulation at 96 h. Here, PPL alone treatment was given at various concentrations ranging from 6.25 to 50 μM. However, differences in the responses have been observed in both the cell lines tested. When HaCaT cells were stimulated with EGF, PPL has shown more reduction in the cell viability which was observed from 3.75 μM concentrations at 48 h time point. Whereas when PPL was treated alone there was no significant change in the cell viability up to 6.25 μM concentration at 96 h time point **(Figure S9a)**. This data suggest that PPL more prominently induces cytotoxicity at growth stimulatory conditions. Our results are also consistent with the previous reports by Roh et al. and Lee et al. that PPL shows less cytotoxicity towards normal cells^8,9^. Apart from this, the effect of PPL on STAT3 phosphorylation was assessed in HaCaT cells at 96 h time point, where we found moderate reduction in the STAT3 phosphorylation at 5 μM concentration, however, a significant reduction was observed at 10 μM concentration **(Figure S9b, c)**.

Additionally, we observed that PPL alone appears to reduce the viability of RAW 264.7 cells significantly from 6.25 μM concentrations **(Figure S10a)**. While, in combination with LPS, PPL showed a reduction in cell viability from 3.75 μM concentration. These differences in the response might be attributed with LPS as previous reports by Xaus et. al and Kuwabara et. al showed that LPS alone has a significant effect on inducing apoptosis signaling^10,11^. We next investigated the effect on p65 phosphorylation at 96 h, we did not find a significant change in the p65 phosphorylation up to 5 μM concentration. However, at 10 μM concentration, a significant decrease in the phosphorylation was observed (**Figure S10b, c)**.

**REFERENCES**

1. Huxford, T., Huang, D. B., Malek, S. & Ghosh, G. The crystal structure of the IkappaBalpha/NF-kappaB complex reveals mechanisms of NF-kappaB inactivation. *Cell* **95**, 759–770 (1998).

2. Watson, P.J., Fairall, L., Santos, G.M. & Schwabe, J.W. Structure of HDAC3 bound to co-repressor and inositol tetraphosphate. *Nature* **481**, 335-40 (2012).

3. **Schrödinger Release 2017-1: P**rotein Preparation Wizard; Epik, Schrödinger, LLC, New York, NY, **2017**; Impact, Schrödinger, LLC, New York, NY, **2017**; Prime, Schrödinger, LLC, New York, NY, **2017**.

4. Halgren, T. A. Identifying and characterizing binding sites and assessing druggability. *J. Chem. Inf. Model* **49**, 377–389 (2009).

5. Halgren, T. New method for fast and accurate binding-site identification and analysis. Chem. Biol. Drug Des. **69**, 146-8 (2007).

6. Friesner, R. A. *et al.* Glide: a new approach for rapid, accurate docking and scoring. 1. Method and assessment of docking accuracy. *J. Med. Chem.* **47**, 1739–1749 (2004).

7. Halgren, T.A. *et al*. Glide: a new approach for rapid, accurate docking and scoring. 2. Enrichment factors in database screening. *J Med Chem*. **47**, 1750-9 (2004).

8. Lee, H.N. *et al.* Heme Oxygenase-1 Determines the Differential Response of Breast Cancer and Normal Cells to Piperlongumine. *Mol Cells* **38**, 327–335 (2015).

9. Roh, J.L., Kim, E.H., Park, J.Y., Kim, J.W., Kwon, M. & Lee BH2. Piperlongumine selectively kills cancer cells and increases cisplatin antitumor activity in head and neck. *Oncotarget* **5**, 9227-38 (2014).

10. Xaus, J. *et al.* LPS induces apoptosis in macrophages mostly through the autocrine production of TNF-alpha. *Blood* **95**, 3823–3831 (2000).

11. Kuwabara, T. & Imajoh-Ohmi, S. LPS-induced apoptosis is dependent upon mitochondrial dysfunction. *Apoptosis* **9**, 467–474 (2004).
