# Supplementary material for: Piperlongumine regulates epigenetic modulation and alleviates psoriasis-like skin inflammation via inhibition of hyperproliferation and inflammation
Source: Cell Death Dis. 2020 Jan 10;11(1):21. doi: 10.1038/s41419-019-2212-y (PMC6954241; doi:10.1038/s41419-019-2212-y)
Supplement: Supplementary file 2 — Supplementary data [file 41419_2019_2212_MOESM2_ESM.docx]

**Table S1.** Average SiteMap values across p65/IκBα protein complex

| **S. No** | **Site name** | **Site score** | **D score** | **Exposure** | **Enclosure** | **Hydrophobic** | **Hydrophilic** |
| --- | --- | --- | --- | --- | --- | --- | --- |
| 1 | Site-I | 1.032 | 0.979 | 0.499 | 0.746 | 0.372 | 1.254 |
| 2 | Site-II | 0.990 | 0.945 | 0.570 | 0.684 | 0.156 | 1.243 |
| 3 | Site-III | 0.995 | 0.984 | 0.528 | 0.690 | 0.390 | 1.136 |
| 4 | Site-IV | 1.041 | 1.076 | 0.722 | 0.723 | 0.498 | 0.913 |
| 5 | Site-V | 0.946 | 0.940 | 0.657 | 0.617 | 0.140 | 1.135 |
