# Supplementary material for: Piperlongumine regulates epigenetic modulation and alleviates psoriasis-like skin inflammation via inhibition of hyperproliferation and inflammation
Source: Cell Death Dis. 2020 Jan 10;11(1):21. doi: 10.1038/s41419-019-2212-y (PMC6954241; doi:10.1038/s41419-019-2212-y)
Supplement: Supplementary file 3 — Supplementary data [file 41419_2019_2212_MOESM3_ESM.docx]

**Table S2.** Average SiteMap values across HDAC3/IκBα protein complex

| **S. No** | **Site name** | **Site score** | **D score** | **Exposure** | **Enclosure** | **Hydrophobic** | **Hydrophilic** |
| --- | --- | --- | --- | --- | --- | --- | --- |
| 1 | Site-I | 1.045 | 1.005 | 0.455 | 0.766 | 0.578 | 1.210 |
| 2 | Site-II | 0.626 | 0.583 | 0.742 | 0.578 | 0.146 | 0.882 |
| 3 | Site-III | 0.598 | 0.563 | 0.765 | 0.591 | 0.411 | 0.652 |
| 4 | Site-IV | 0.597 | 0.298 | 0.641 | 0.612 | 0.000 | 1.739 |
