# Supplementary material for: Piperlongumine regulates epigenetic modulation and alleviates psoriasis-like skin inflammation via inhibition of hyperproliferation and inflammation
Source: Cell Death Dis. 2020 Jan 10;11(1):21. doi: 10.1038/s41419-019-2212-y (PMC6954241; doi:10.1038/s41419-019-2212-y)
Supplement: Supplementary file 4 — Supplementary data [file 41419_2019_2212_MOESM4_ESM.docx]

**Table S3**. GLIDE docking results and Prime MM/GBSA binding energy calculations for PPL at the binding sites of p65/IκBα and HDAC3/IκBα protein complexes, respectively.

| **Ligand**  **name** | **Targeted protein** | **Docking score** | **Binding energy**  **(kcal*/*mol)** | **Interactions** | | |
| --- | --- | --- | --- | --- | --- | --- |
|  |  |  |  | **H- bonds** | **π – π** | **Hydrophobic** |
| PPL | p65/IκBα | -4.365 | -21.321 | Arg143, Asn145, Arg253 | His184, Arg253 | Leu189, Ile192, Cys215, Leu223, Leu227 |
|  | HDAC3/IκBα | -6.783 | -60.613 | Cys167, Val300, Arg301 | - | Leu130, Leu131, Ala133, Cys167, Cys263, Tyr298, Val300, Tyr331 |
