# Supplementary material for: Piperlongumine regulates epigenetic modulation and alleviates psoriasis-like skin inflammation via inhibition of hyperproliferation and inflammation
Source: Cell Death Dis. 2020 Jan 10;11(1):21. doi: 10.1038/s41419-019-2212-y (PMC6954241; doi:10.1038/s41419-019-2212-y)
Supplement: Supplementary file 5 — Supplementary data [file 41419_2019_2212_MOESM5_ESM.docx]

**Supplementary Figure Legends**

**Figure S1. Rheological studies of piperlongumine (PPL) topical gel.** Various concentrations of carbapol gels (0.1-0.75%) were prepared and rheological properties were determined by modular compact rheometer (Anton-Paar, USA) and evaluated the **(a)** shear stress vs shear rate and **(b)** viscosity vs shear rate.

**Figure S2.** **Piperlongumine (PPL) alone treatment on the skin compliance and safety evaluation. (a)** Representative images of BALB/c mice from control (without treatment), PPL alone topically at 30 mg/kg (PH) and PPL alone subcutaneous administration (PSC) on the 7^th^ day of animal sacrifice to observe the phenotypic changes associated with PPL treatment. (**b)** H&E staining was performed on the skin tissue sections to monitor the pathological changes associated with the treatment (×400 magnification). **(c)** Epidermal thickness was measured by Nikon NIS elements Software. (**d)** Immunoblotting was performed in skin tissues to evaluate the effect of PPL treatment on phosphorylation of STAT3 and p65 after 7 days. **(e, f)** Blots intensity was quantified by Image J analysis. The data represents the mean ± SD (n=3 independent experiments).

**Figure S3.** **Piperlongumine (PPL) alone treatment evaluation on the skin compliance. (a)** Representative images of BALB/c mice were captured from control (without treatment), PPL alone topically at 30 mg/kg (PH) and PPL alone subcutaneous administration (PSC) on the 21st day of animal sacrifice to observe the phenotypic changes associated with PPL treatment. (**b)** H & E staining was performed on the skin tissue sections to monitor the pathological changes associated with the treatment (×400 magnification). **(c)** Epidermal thickness was measured by Nikon NIS elements Software. (**d)** Immunoblotting was performed in skin tissues to evaluate the effect of PPL treatment on phosphorylation of STAT3 and p65 after 21 days. (**e, f**) Blots intensity was quantified by Image J analysis. The data represents the mean ± SD (n=3 independent experiments).

**Figure S4. PPL intervention ameliorates Imiquimod (IMQ) induced psoriasis like skin inflammation.** **(a)** PPL treatment attenuated the IMQ induced splenomegaly. Spleen mass was calculated by body mass index (spleen weight alone/total body weight) in all the mentioned groups and compared with IMQ control. Similarly, **(b)** ear thickness was measured every alternate day from day 0 up to termination by vernier calipers. Epidermal thickness induced by IMQ was measured by Nikon NIS elements Software at **(c)** ×200 and **(d)** ×400. Immunoblotting shows the effect of PPL on the **(e-h)** mTOR expression, phosphorylation of AKT at Ser474, Thr308 sites and p70S6kinase. **(i-l)** The expression of proliferation markers Ki67, PCNA, Cyclin D1, and Bcl-2 was determined by immunoblotting. Data represents as mean ± SD (n=5 mice per group). ***P*<0.0, ****P*<0.001 and *****P*<0.0001 are significantly different from the NC group. ^^^*P*<0.05, ^^^^*P*<0.01, ^^^^^*P*<0.001, and ^^^^^^*P*<0.0001 are significantly different from the IMQ group. Here, PL=PPL 10 mg/kg and PH=PPL 30 mg/kg topically, PSC=PPL 1 mg/kg subcutaneously, and TAC=Tacrolimus 20 mg/kg topically.

**Figure S5. PPL reduces hyperproliferation of keratinocytes with induction of apoptosis.** HaCaT cells were pre-treated with PPL at two-fold serial dilutions ranging from 1.87 to 15 μM. After 2 h, cells were stimulated with EGF (50 ng/ml), except normal control (NC) and incubated for **(a)** 24 and **(b)** 48 h, respectively and cell viability was assessed by MTT assay. **(c)** The bar chart depicts the quantification of PPL of cell cycle phases (Sub G1, G0/G1, S and G2-M distribution. Flow cytometric analysis was performed to determine **(d)** the loss of mitochondrial membrane potential (ΔΨm). **(d)** The early or late apoptosis was determined by Annexin V Alexa flour 488/propidium iodide staining. Western blotting analysis was performed to determine the effect of PPL on molecular protein expression of **(f)** mTOR and **(g-i)** the phosphorylation of AKT at ser474, Thr308 sites including p70S6 kinase**. (j-m)** The expression of proliferation markers includes Ki67, PCNA, Cyclin D1 and Bcl-2 were determined by western blotting. **(n)** Apoptotic induction was quantified by increase in the TUNEL positivity measured by Fiji-Image j. **(o)** DCFDA staining was performed to determine the ROS generation by PPL at 5 µM, under the EGF stimulation followed by oxidative stress (300 μM H_2_O_2_) conditions. The data represents the mean ± SD (n=3). ****P*<0.001, and *****P*<0.0001 vs. NC. ^*P*<0.05, ^^*P*<0.01, ^^^*P*<0.001, and ^^^^*P*<0.0001 are significantly different from the EGF control.

**Figure S6.** **PPL treatment reduces the expression of keratin 17 (K17) by inhibiting STAT3 expression.** Western blot analysis was performed to determine the expression of p-STAT3 at Tyr705, p-ERK1/2 and K17 expression by PPL treatment in **(a-c)** HaCaT cells and **(d-f)** skin tissues. The blots were quantified by image j analysis. Immunofluorescence for the expression of (**g**) K17 and (**h**) STAT3 from skin tissue sections was quantified by Fiji-Image J software. The data represents the mean ± SD (n=3 independent experiments, *in vitro*; n=5 animals per group, *in vivo)* **P*<0.05, ***P*<0.01, ****P*<0.001, and *****P*<0.0001 vs. NC. ^*P*<0.05, ^^*P*<0.01, ^^^*P*<0.001, and ^^^^*P*<0.0001 are significantly different from the EGF/IMQ group. Here, PL=PPL 10 mg/kg and PH=PPL 30 mg/kg topically, PSC=PPL 1 mg/kg subcutaneously, and TAC=Tacrolimus 20 mg/kg topically.

**Figure S7. PPL inhibits the protein expression of the inflammatory signaling cascade and exerts potent HDAC inhibitory activity in murine macrophages and skin tissues.** NF-κB signaling pathway proteins such as ICAM1 and COX-2 proteins expression were determined upon PPL treatment in **(a-g)** macrophages and **(h-n)** skin tissues**.** Similarly, class-I epigenetic regulated markers (HDACs 1-4) expression from nuclear extracts which were normalized by H3, while class II HDAC6 expression was determined from the whole cell extract which was normalized by β-Actin were analyzed in **(o-s)** RAW 264.7 cells and **(t-x)** skin tissues**.** The data represents the mean ± SD (n=3 independent experiments, *in vitro*; n=5 animals per group, *in vivo).***P*<0.05, ***P*<0.01, ****P*<0.001, and *****P*<0.0001 vs. NC. ^^^*P*<0.05, ^^*P*<0.01, ^^^*P*<0.001, and ^^^^*P*<0.0001 are significantly different from the LPS/IMQ group. Here, PL=PPL 10 mg/kg and PH=PPL 30 mg/kg topically, PSC=PPL 1 mg/kg subcutaneously, and TAC=Tacrolimus 20 mg/kg topically.

**Figure S8. PPL potently inhibits the** **nuclear translocation of p65 and HDAC3 induced by LPS.** RAW 264.7 cells were pre-treated with PPL at indicated concentrations. After 12 h, cells were stimulated with LPS (1 µg/ml) and incubated for 30 min, both **(a, b)** cytosolic and **(c, d)** nuclear proteins were isolated. p65 and HDAC3 proteins expression from cytosolic and nuclear fractions from immunoblotting analysis were quantified by Image j analysis. Similarly, the expressions of (**e**) p65 and (**f**) HDAC3 were analyzed by confocal microscope and quantified in skin tissue sections. The data represents the mean ± SD (n=3 independent experiments, *in vitro*; n=5 animals per group, *in vivo).* ****P*<0.001 and *****P*<0.0001 vs. NC. ^*P*<0.05 ^^*P*<0.01, ^^^*P*<0.001, and ^^^^*P*<0.0001 are significantly different from the EGF/IMQ group. Here, PL=PPL 10 mg/kg and PH=PPL 30 mg/kg topically, PSC=PPL 1 mg/kg subcutaneously, and TAC=Tacrolimus 20 mg/kg topically.

**Figure S9. Effect of PPL alone treatment on HaCaT cells. (a)** HaCaT cells were treated with PPL at concentrations ranging from 6.25 to 50 μM concentrations and incubated for 96 h, **(b)** The phosphorylation of STAT3 at Tyr 705 was analyzed at 96 h time point with PPL alone at various concentrations 1, 2.5, 5, 7.5 and 10 μM and further subjected to immunobloting. **(c)** The phosphorylation of STAT3 at Tyr 705 was quantified by Image J densitometric analysis and normalized with STAT3.The data represents the mean ± SD (n=3 independent experiments) **P*<0.05, ****P*<0.001, and *****P*<0.0001 vs Control.

**Figure S10. Effect of PPL alone treatment on RAW 264.7 cells. (a)** Raw 264.7 cells were treated with PPL at concentrations ranging from 6.25 to 50 μM concentrations and incubated for 96 h, (**b)** The phosphorylation of p65 was analyzed at 96 h time point with PPL alone at various concentrations 1, 2.5, 5, 7.5, and 10 μM and further subjected to immunoblotting. (**c)** The phosphorylation of p65 was quantified by Image J densitometric analysis and normalized with p65. The data represents the mean ± SD (n=3 independent experiments, **P*<0.05, ***P*<0.01 and *****P*<0.0001 vs Control.
