# Supplementary figures and images for: Piperlongumine regulates epigenetic modulation and alleviates psoriasis-like skin inflammation via inhibition of hyperproliferation and inflammation
Source: Cell Death Dis. 2020 Jan 10;11(1):21. doi: 10.1038/s41419-019-2212-y (PMC6954241; doi:10.1038/s41419-019-2212-y)

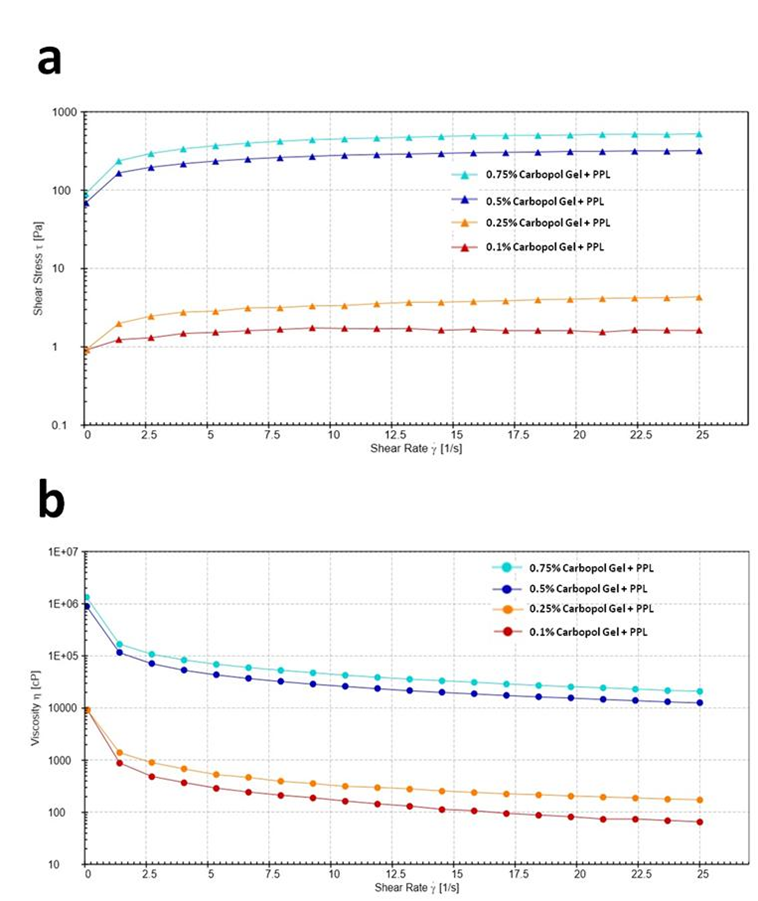

Supplement: Supplementary file 6 — Supplementary data [file 41419_2019_2212_MOESM6_ESM.tif]

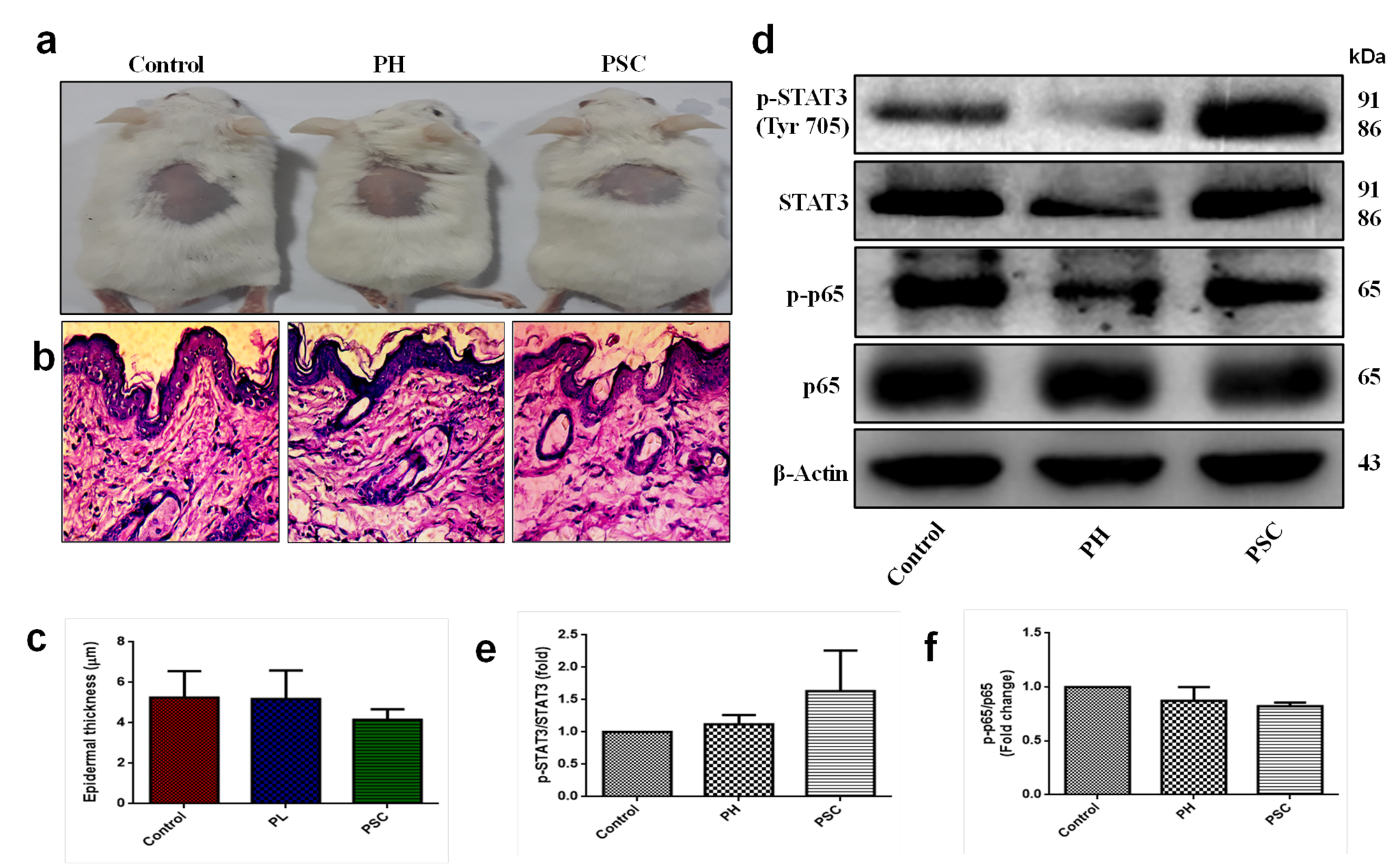

Supplement: Supplementary file 7 — Supplementary data [file 41419_2019_2212_MOESM7_ESM.tif]

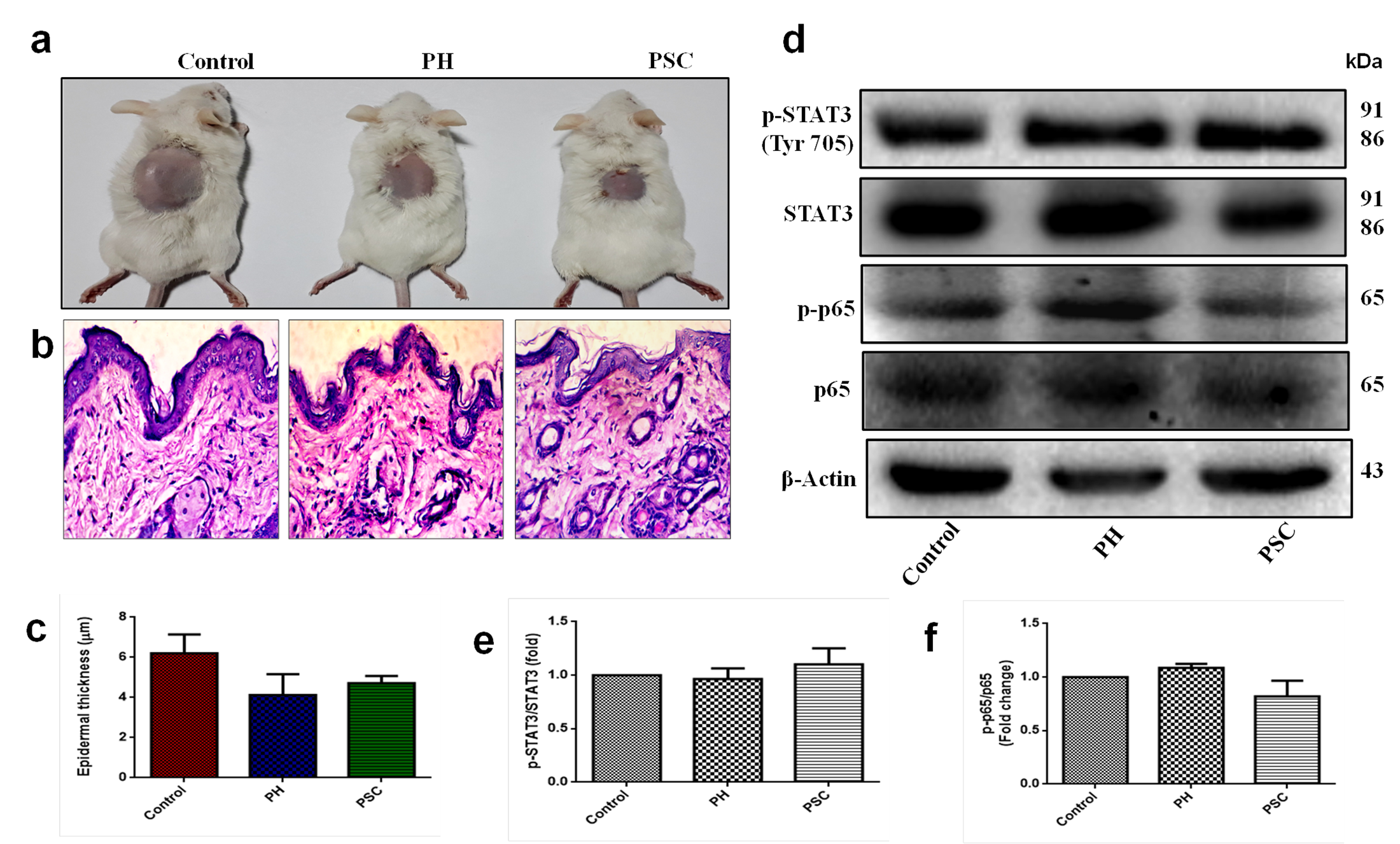

Supplement: Supplementary file 8 — Supplementary data [file 41419_2019_2212_MOESM8_ESM.tif]

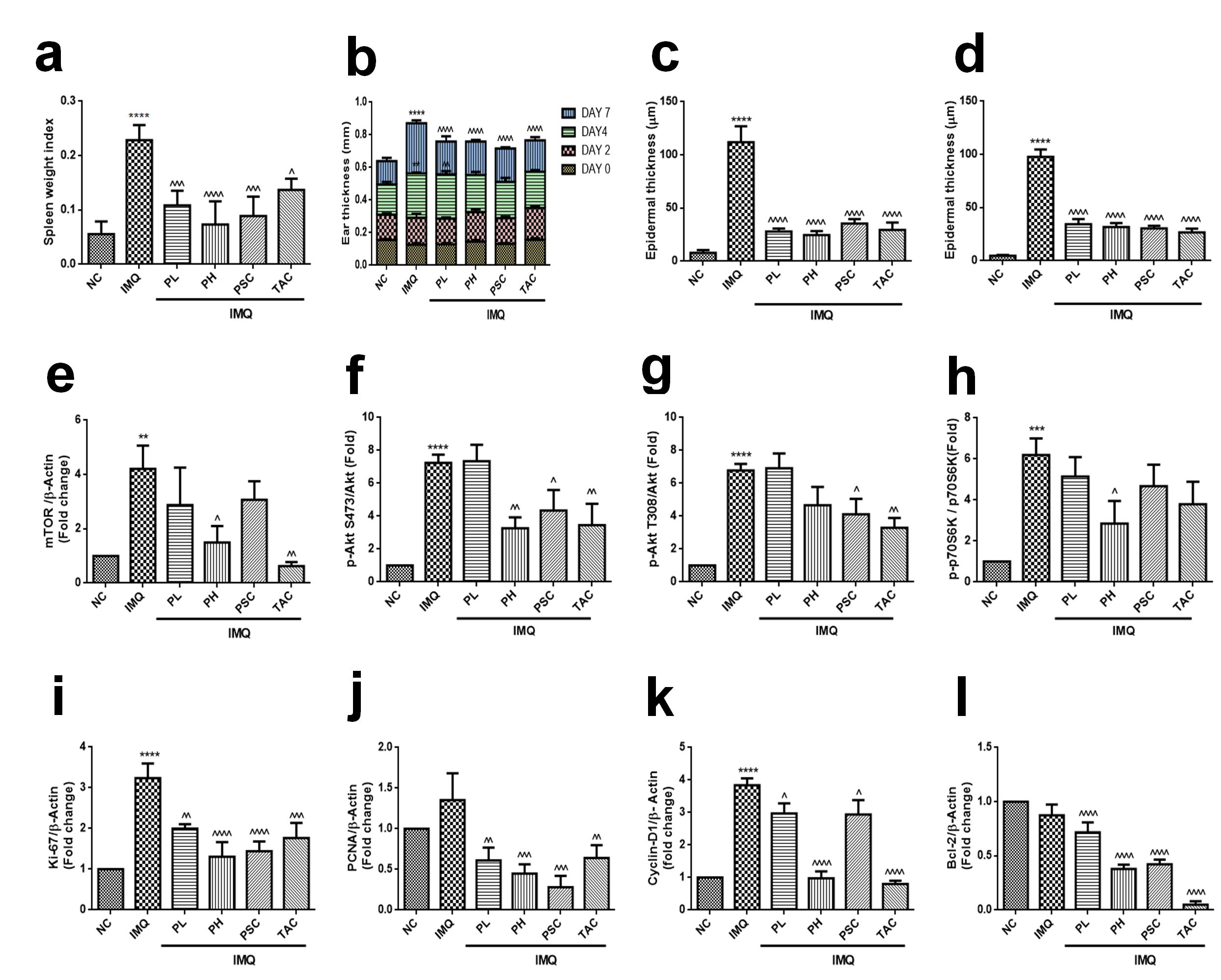

Supplement: Supplementary file 9 — Supplementary data [file 41419_2019_2212_MOESM9_ESM.tif]

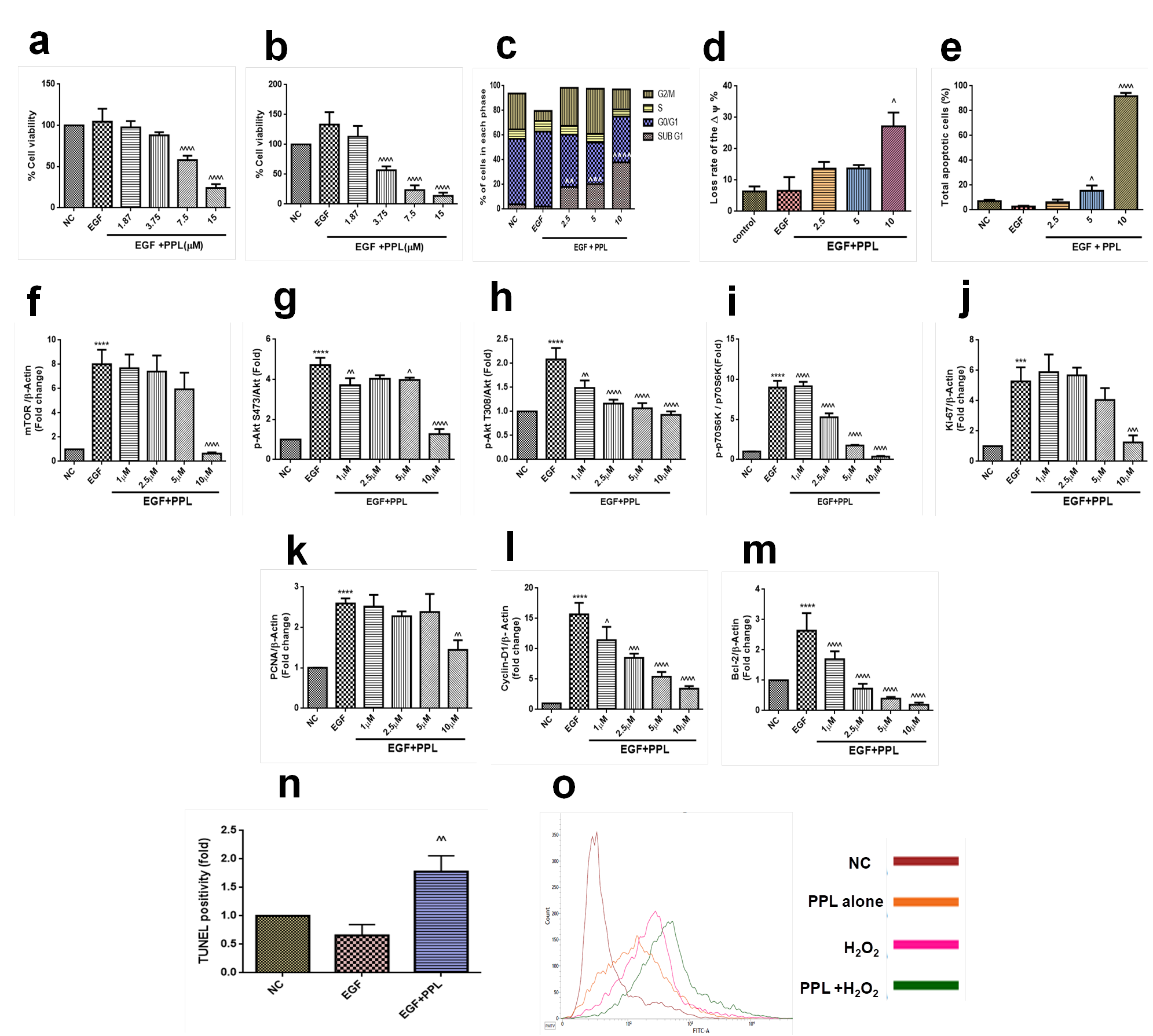

Supplement: Supplementary file 10 — Supplementary data [file 41419_2019_2212_MOESM10_ESM.tif]

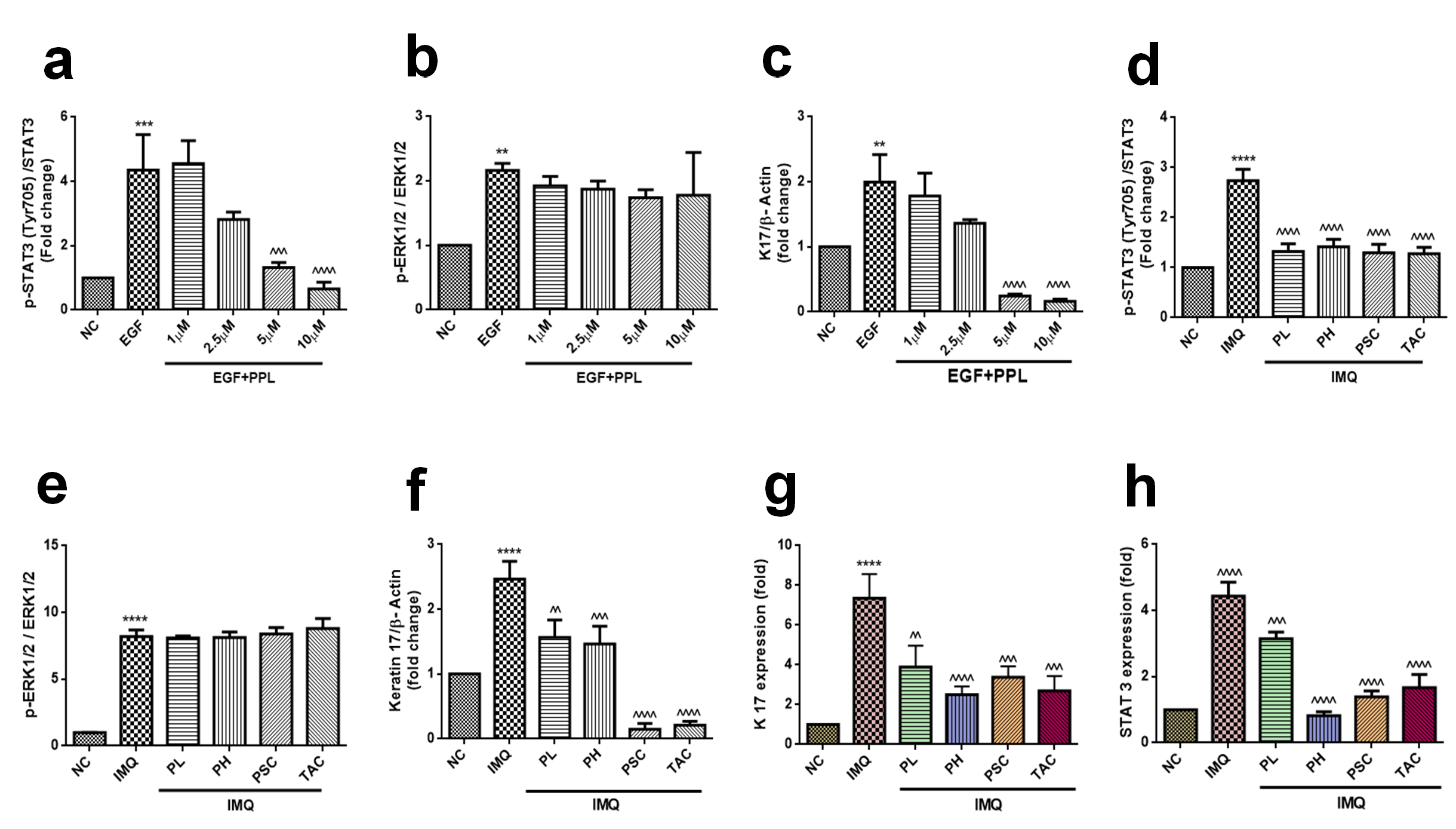

Supplement: Supplementary file 11 — Supplementary data [file 41419_2019_2212_MOESM11_ESM.tif]

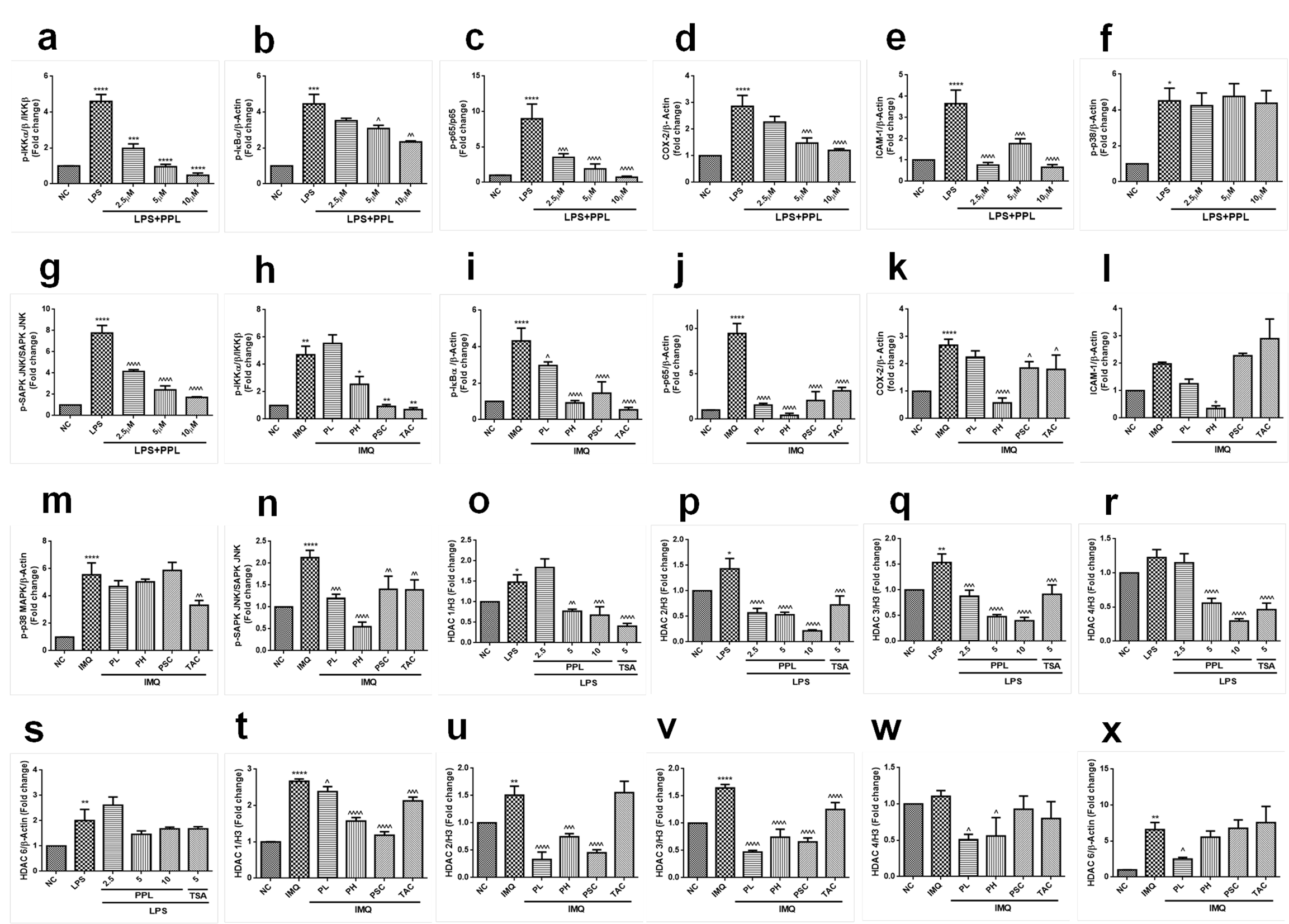

Supplement: Supplementary file 12 — Supplementary data [file 41419_2019_2212_MOESM12_ESM.tif]

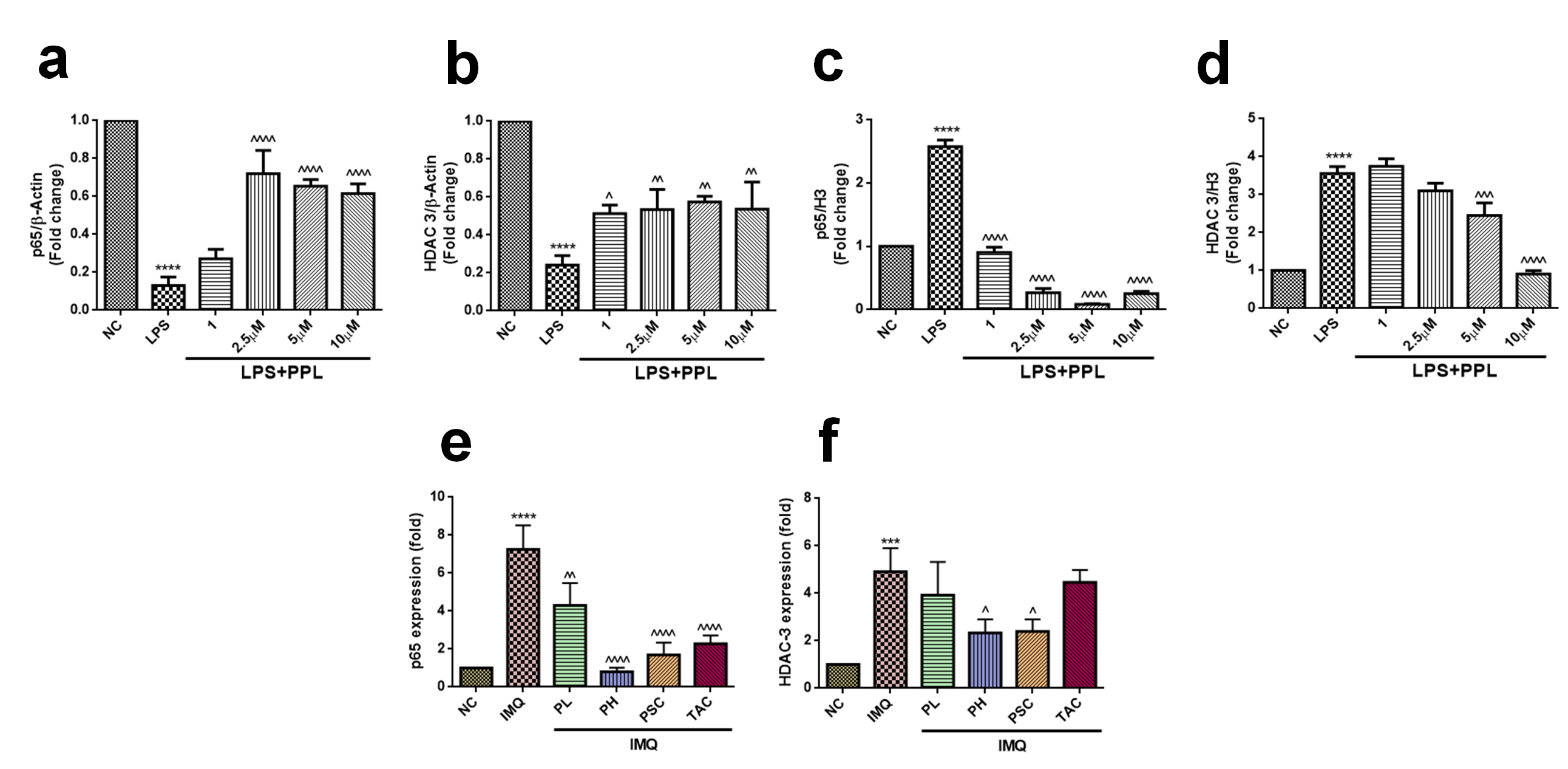

Supplement: Supplementary file 13 — Supplementary data [file 41419_2019_2212_MOESM13_ESM.tif]

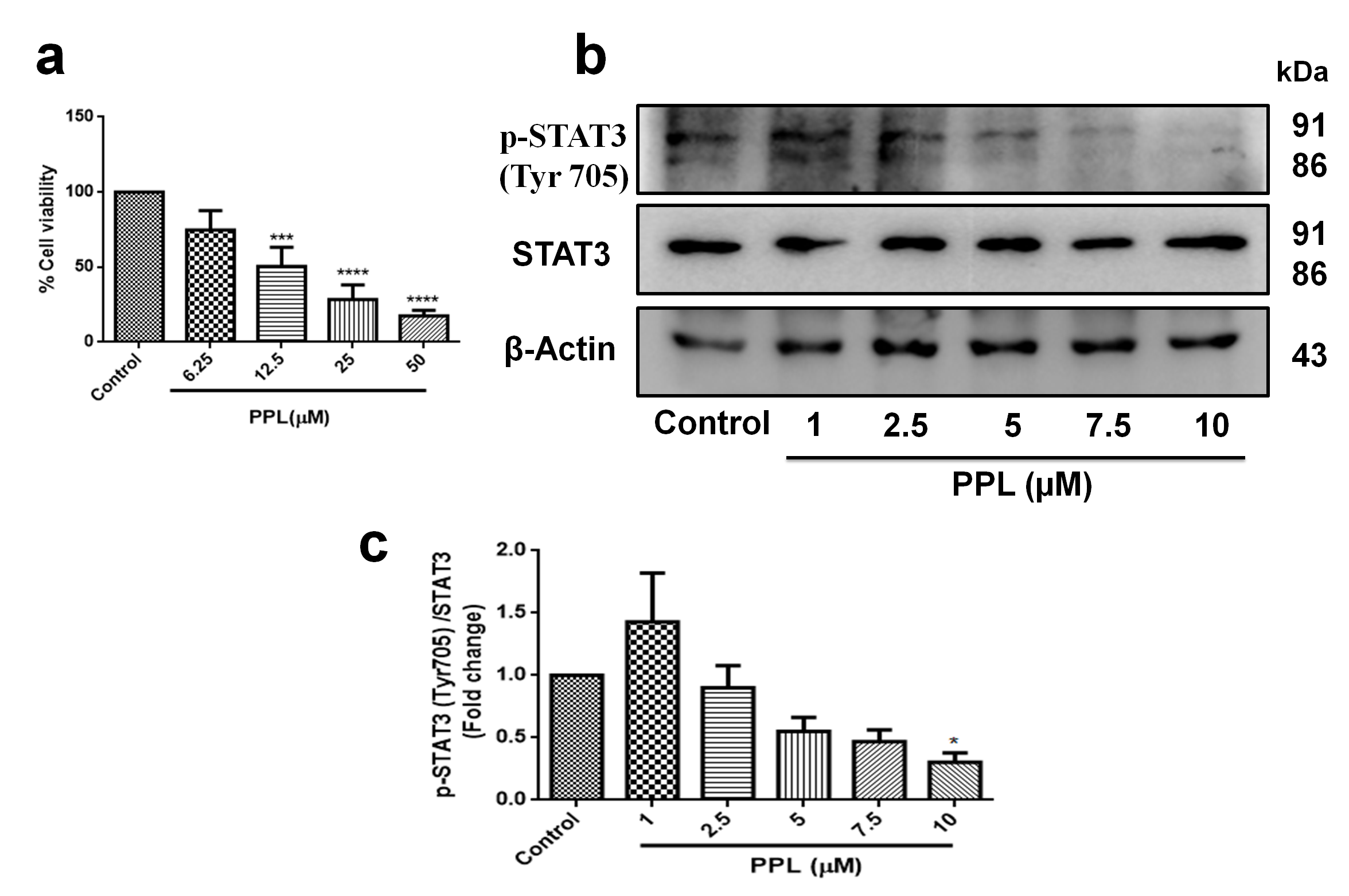

Supplement: Supplementary file 14 — Supplementary data [file 41419_2019_2212_MOESM14_ESM.tif]

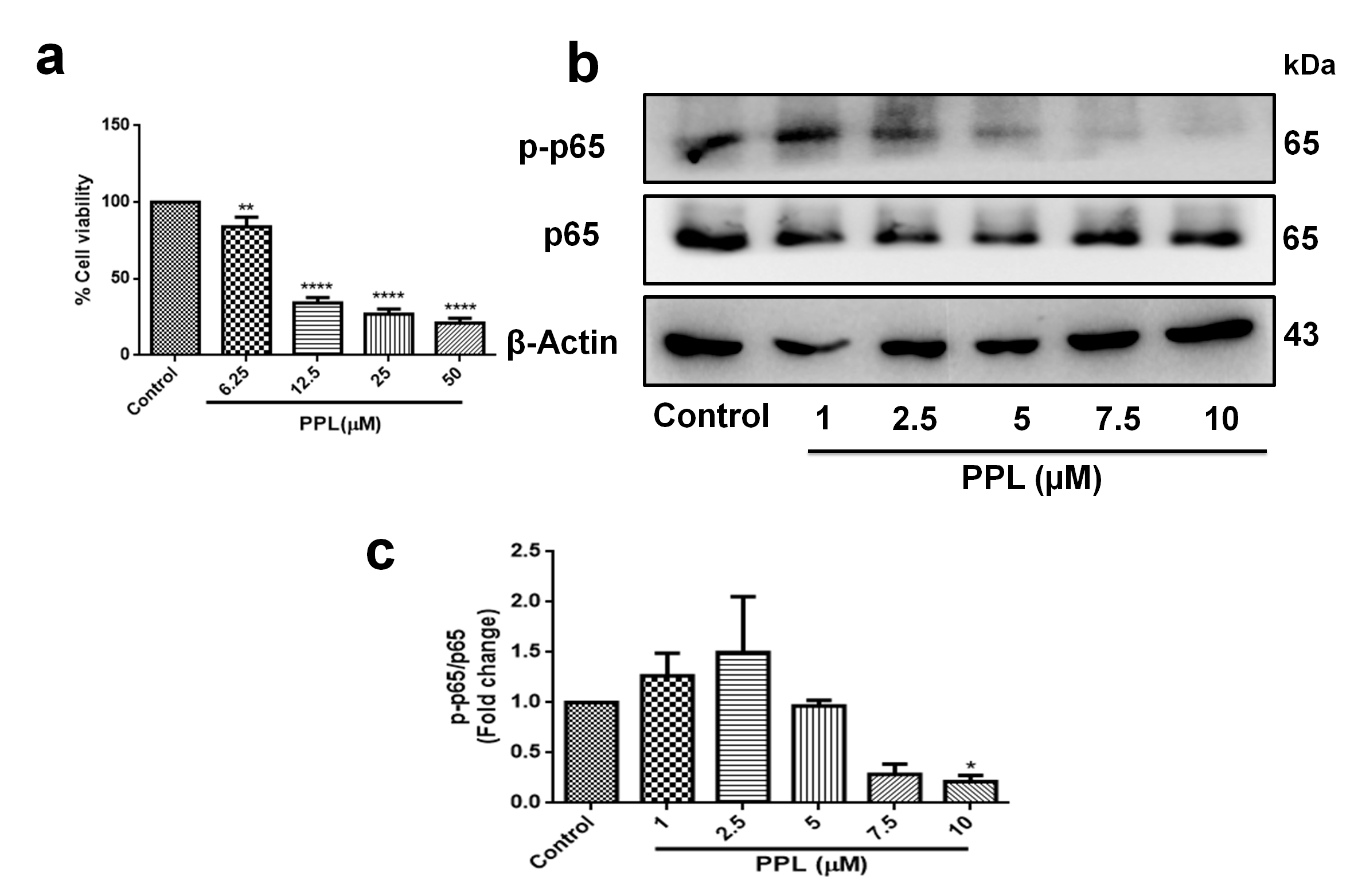

Supplement: Supplementary file 15 — Supplementary data [file 41419_2019_2212_MOESM15_ESM.tif]
